# LncRNAs2Pathways: Identifying the pathways influenced by a set of lncRNAs of interest based on a global network propagation method

**DOI:** 10.1038/srep46566

**Published:** 2017-04-20

**Authors:** Junwei Han, Siyao Liu, Zeguo Sun, Yunpeng Zhang, Fan Zhang, Chunlong Zhang, Desi Shang, Haixiu Yang, Fei Su, Yanjun Xu, Chunquan Li, Huan Ren, Xia Li

**Affiliations:** 1 College of Bioinformatics Science and Technology, Harbin Medical University, Harbin, 150081, P. R. China; 2 School of Medical Informatics, Daqing Campus, Harbin Medical University, Harbin, 150081, P. R. China; 3 Department of immunology, Harbin Medical University, Harbin 150081, P. R. China

## Abstract

Long non-coding RNAs (lncRNAs) have been demonstrated to play essential roles in diverse cellular processes and biological functions. Exploring the functions associated with lncRNAs may help provide insight into their underlying biological mechanisms. The current methods primarily focus on investigating the functions of individual lncRNAs; however, essential biological functions may be affected by the combinatorial effects of multiple lncRNAs. Here, we have developed a novel computational method, LncRNAs2Pathways, to identify the functional pathways influenced by the combinatorial effects of a set of lncRNAs of interest based on a global network propagation algorithm. A new Kolmogorov–Smirnov-like statistical measure weighted by the network propagation score, which considers the expression correlation among lncRNAs and coding genes, was used to evaluate the biological pathways influenced by the lncRNAs of interest. We have described the LncRNAs2Pathways methodology and illustrated its effectiveness by analyzing three lncRNA sets associated with glioma, prostate and pancreatic cancers. We further analyzed the reproducibility and robustness and compared our results with those of two other methods. Based on these analyses, we showed that LncRNAs2Pathways can effectively identify the functional pathways associated with lncRNA sets. Finally, we implemented this method as a freely available R-based tool.

Long non-coding RNAs (lncRNAs) are non-protein-coding transcripts >200 nucleotides that have been reported to play essential roles in diverse cellular processes and biological functions, such as transcriptional regulation, chromatin modification, cell differentiation, epigenetic regulation and immune responses^1^,^2^,^3^,^4^,^5^. More importantly, emerging evidence suggests that the dysregulation of lncRNAs is associated with the development and progression of a variety of human diseases, including cancer and other immune and neurological disorders^6^,^7^,^8^. Therefore, exploring the biological functions influenced by lncRNAs may help provide insight into the underlying mechanisms of lncRNAs in human diseases. With the development of next-generation sequencing technologies, tens of thousands of lncRNAs have been identified. As lncRNAs are generally weakly conserved in their primary sequences and interaction data between lncRNAs and other molecules are lacking^9^, the functions of most lncRNAs in complex diseases have not been widely studied.

Several recent methods of lncRNA function identification have been proposed. These methods can be classified into experimental and computational methods^9^. Although experimental methods can give more reliable lncRNA functions than computational methods, they are expensive and time consuming. Computational analysis to identify the probable functions of lncRNAs is promising as a way to guide further studies on lncRNAs. Guttman *et al*.^10^ assigned functions to ~1600 mouse long-intervening non-coding RNAs (lincRNAs) identified by chromatin-state maps based on coding–non-coding gene co-expression relationships extracted from custom-designed tiling array data. Liao *et al*.^11^ constructed a coding–non-coding gene co-expression network from re-annotated mouse microarray data and predicted the functions of 340 mouse lncRNAs based on topological or other network characteristics. Guo *et al*.^12^ developed a lncRNA global function predictor by integrating coding–non-coding co-expression data and protein interaction data. In this method, an information flow algorithm was applied, and 1625 mouse lncRNAs were functionally characterized. The LncRNAdb database included comprehensive functions for 280 eukaryotic cell lncRNAs by collecting curated literature evidence^13^. Linc2Go was developed as a functional annotation resource for lincRNA^14^. It integrated microRNA-mRNA and microRNA-lincRNA interaction data to generate comprehensive functional annotations for human long intergenic non-coding RNA based on the competing endogenous RNA hypothesis. The LncRNAtor platform collected 208 RNA-Seq datasets and identified co-expressed protein-coding genes of lncRNAs, which were then subjected to functional enrichment analysis^15^. Despite accumulating insights into lncRNA functions, the above studies primarily focused on investigating individual lncRNA functions and did not consider the combinatorial effects of multiple lncRNAs. However, recent evidence has demonstrated that essential cellular processes and biological functions may be affected by a set of lncRNAs, such as differentially expressed lncRNAs between cancer and normal samples^16^,^17^.

To identify the important functional terms affected by a set of lncRNAs, Jiang *et al*.^18^ developed a web interface named LncRNA2Function. LncRNA2Function first investigates the expression correlation between lncRNAs and protein-coding genes across the RNA-Seq data of 19 human normal tissues and then performs the hypergeometric test to functionally annotate a set of lncRNAs with significantly enriched functional terms among the protein-coding genes co-expressed with the lncRNAs. Zhao *et al*.^19^ introduced Co-LncRNA, a web-based computational tool that provides enrichment analyses of lncRNAs for Gene Ontology (GO) annotations and Kyoto Encyclopedia of Genes and Genomes (KEGG) pathways. Co-LncRNA collects 241 publicly available human RNA-Seq datasets and identifies the co-expressed protein-coding genes associated with multiple lncRNAs. The combinatorial effects of lncRNAs in the modulation of a given functional term are investigated by the simultaneous analysis of multiple lncRNAs. Although both LncRNA2Function and Co-LncRNA helped us to explore the combinatorial effects of a set of lncRNAs, only directly co-expressed protein-coding genes of lncRNAs were exploited, and their downstream genes were neglected. Typically, protein-coding and non-coding genes co-operate as a biological system. The network-based strategy has been successfully used for protein function annotation^20^ and tumor biomarker identification^21^, and thus, this strategy may be a promising way to address the combinatorial effects of a set of lncRNAs.

Here, we developed a novel computational method, LncRNAs2Pathways, to identify the functional pathways influenced by the combinatorial effects of a set of interesting lncRNAs based on a global network propagation algorithm. In this study, we first used 28 independent RNA-Seq datasets under different experimental conditions to extract gene co-expressed relationships of coding–coding, coding–non-coding and non-coding–non-coding. The co-expressed relationships were integrated with protein–protein interaction data to construct a coding–non-coding gene correlation (CNC) network, in which the nodes represent protein-coding and non-coding genes, and the edges are co-expression and protein–protein interactions. We then obtained a set of interesting lncRNAs, such as differentially expressed lncRNAs between disease and normal samples, and mapped them to the CNC network as source nodes. When a protein-coding gene is located closer to the lncRNAs, the gene may be more likely to be regulated. We used a global network propagation algorithm, random walk with restart (RWR), to calculate the propagation scores of protein-coding genes, which reflect the extent of genes influenced by the lncRNAs. A list of protein-coding genes was formed by ranking the protein-coding genes according to their propagation scores. Finally, we used a new Kolmogorov–Smirnov-like statistical measure weighted by the propagation scores to evaluate each pathway by mapping the genes in the pathway to the ranked gene list. We applied LncRNAs2Pathways to three sets of lncRNAs associated with prostate cancer, glioma, and pancreatic cancer. We then analyzed the reproducibility and robustness and compared our method with two other function analysis methods for lncRNAs. Our results indicate that LncRNAs2Pathways can produce biologically meaningful outcomes.

## Materials and Methods

**Figure 1:**
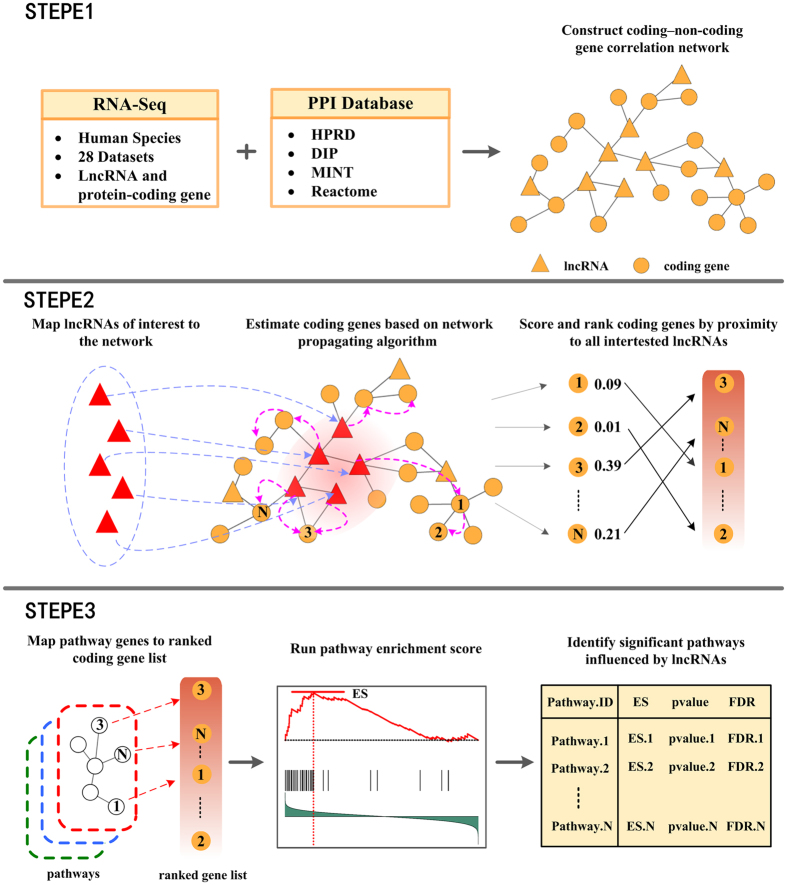
Flow diagram of LncRNAs2Pathways. Step 1. RNA-Seq data and protein–protein interaction data are integrated to construct a CNC network. Step 2. A set of lncRNAs of interest are mapped to the gene correlation network, and the global network propagation algorithm is used to calculate the propagation scores of protein-coding genes, which reflect the extent of the genes influenced by the lncRNAs. A ranked protein-coding gene list is constructed according to the propagation scores. Step 3. Protein-coding genes in a given pathway are mapped to the ranked protein-coding gene list, and the *ES(P*) is calculated by walking down the list. The permutation test is performed to identify the statistically significant pathways.

LncRNAs2Pathways was developed to identify the biological pathways influenced by the combinatorial effects of a set of lncRNAs of interest. Figure 1 depicts the flow diagram of the LncRNAs2Pathways. The main steps consist of (1) constructing the CNC network by integrating RNA-Seq data and protein–protein interaction data, (2) estimating the extent of protein-coding genes influenced by the set of lncRNAs of interest based on a global network propagation algorithm, and (3) calculating pathway enrichment scores (*ES*s) to evaluate the biological pathways. LncRNAs2Pathways has been implemented as a freely available R-based tool (https://cran.r-project.org/web/packages/LncPath/). The user inputs a set of lncRNAs of interest, and the biological pathways influenced by the lncRNAs are then returned.

### Constructing the CNC network

We collected 28 human RNA-Seq datasets covering a wide range of experimental and physiological conditions from the National Center for Biotechnology Information (NCBI) Sequence Read Archive (SRA) database^22^ (see Supplementary Table S1 for the list of datasets). Each dataset was required to contain at least six samples. For each dataset, RNA-Seq reads were mapped to the human genome by TopHat v2.0.13^23^, and the expression values of human lncRNAs and protein-coding genes were quantified by Cufflinks v2.2.1^24^. Both TopHat and Cufflinks were performed with the default parameters. The gene expression values were measured as fragments per kilobase of exon per million fragments mapped (FPKM). For this study, annotations of all human lncRNAs and protein-coding genes were downloaded from GENCODE v22^25^.

We then extracted coding–coding, coding–non-coding and non-coding–non-coding gene co-expression relationships based on the 28 RNA-Seq datasets. For each dataset, the data were used as follows:

1. Genes (lncRNAs and protein-coding genes) with an average FPKM >1^15^ and the variance of FPKM ranked in the top 75% of all genes in the dataset were retained^11^.
2. The Pearson correlation coefficient (Pcc) between the FPKM values of each gene pair was calculated. We applied Fisher’s *z* transform method^26^ to convert each correlation coefficient *r* to the *z*-statistic by *z* = 0.5[ln(1 + *r*)−ln(1−*r*)]. The result was approximately normal with a standard error of 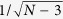, where *N* is the sample size of the dataset. For each gene, we standardized the *z*-scores to enforce zero mean and unit variance, and then, a set of Pcc *p*-values was calculated for each gene pair. Bonferroni multiple test correction was implemented to adjust the *p*-values.
3. Gene pairs in the given dataset were considered co-expressed if they had a Pcc value ranked in the top or bottom 0.1% for each gene and an adjusted *p*-value < 0.01.

According to the sign of the Pcc, gene co-expression relationships can be classified as positive correlation or negative correlation (Pcc > 0 or Pcc < 0). To derive reliable co-expressed relationships, we only retained co-expressed gene pairs with the same correlation direction (i.e., positive or negative) in three or more datasets. Thus, 114,006 co-expression relationships of coding–coding, coding–lncRNA and lncRNA–lncRNA genes were obtained (Supplementary Table S2).

To model real and comprehensive biological processes, we further integrated gene co-expression relationships with protein–protein interactions. Protein interaction data for humans were downloaded from four public available databases (Human Protein Reference Database [HPRD]^27^, the Database of Interacting Proteins [DIP]^28^, the Molecular INTeraction database [MINT]^29^ and Reactome^30^). We merged gene co-expression relationships with protein–protein interactions to construct a CNC network. The CNC network consists of 28,613 nodes (11,391 lncRNAs and 17,222 protein-coding genes) and 295,698 edges (104,391 gene co-expressions, 181,692 protein–protein interactions and 9,615 both gene co-expression and protein–protein interaction) (see Supplementary Table S2 for details about the CNC network). By detecting the degree distribution of the CNC network (Supplementary Figure S1), we found that it follows a power law distribution (*P(k*)～*k*^−*γ*^, *γ* = 1.71 and fitted line R-squared = 0.9185), which means that it is a scale-free network.

### Estimating the extent of protein-coding genes influenced by lncRNAs of interest

We mapped a set of lncRNAs of interest, such as differentially expressed lncRNAs between disease and normal samples, to the CNC network as source nodes. The CNC network was constructed based on gene co-expression and protein–protein interaction, and the closer a protein-coding gene is located to the source nodes, the more likely this gene may be to be regulated. We used a global network propagation algorithm, RWR, to estimate the extent of protein-coding genes influenced by the source nodes. The RWR algorithm^31^ simulates an iterative walker that transitions from its current source node (or a set of source nodes simultaneously) to a randomly selected neighbor or returns to the source node(s) with a given probability. It can be used to compute the network-proximity of a node to the source node(s). This algorithm has been used to prioritize candidate disease genes^32^,^33^. Here, we applied the RWR algorithm to prioritize the protein-coding genes influenced by lncRNAs of interest in the CNC network. Formally, the RWR is defined as follows:


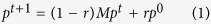


where *M* is the column-normalized adjacency matrix of the CNC network graph; *p*^*t*^ is the vector of nodes at time step *t*, and its *i*-th element holds the probability of being at node *i* at time step *t*; and *p*^0^ is the initial probability vector of nodes, which was constructed by assigning to source nodes with 1 and other nodes with 0 and then normalized to a unit vector. This is equivalent to letting the random walker begin from each source node with equal probability. The parameter *r* is the restart probability of the walk in every time step at the source nodes. Kohler *et al*.^32^ proposed that *r* has only a slight effect on the results of the RWR algorithm when *r* varies from 0.1 to 0.9. In this study, the parameter *r* was set at 0.7.

The probability vector *p*^*t*^ will converge to a unique steady state *p*^*∞*^after certain steps. This was achieved at query time by iterating until the *L*_1_-norm between *p*^*t*^ and *p*^*t+*1^ fell below 10^−10^. *p*^*∞*^provides a measure of the extent of the genes in the CNC network influenced by the source nodes. At the steady state *p*^*∞*^, the protein-coding genes were assigned with the probabilities at their corresponding nodes in the CNC network graph. A protein-coding gene with a larger probability indicates that the gene locates closer to the source nodes and, thus, may be influenced by the source nodes to a greater extent. We then normalized the probabilities of protein-coding genes to their square roots and defined them as *propagation scores*. Finally, a protein-coding gene list *L* = < *g*_1_, *g*_2_,… *g*_n_> was generated by ranking the protein-coding genes in the CNC network according to their *propagation scores*.

### Calculating pathway *ES*s to evaluate the biological pathways

We downloaded pathways from the KEGG databases^34^,^35^. Pathways with fewer than 15 protein-coding genes or more than 500 protein-coding genes in the CNC network were filtered out to avoid overly narrow or broad functional categories.

For each pathway, we mapped the protein-coding genes to the ranked gene list *L*. If the protein-coding genes in the pathway occur toward the top of the list *L*, the pathway will tend to be influenced by the combinatorial effects of the inputted lncRNAs. Inspired by Gene Set Enrichment Analysis (GSEA)^36^, we used a Kolmogorov–Smirnov-like statistic weighted by the *propagation score* to calculate an *ES*, which reflects the degree to which the pathway is overrepresented at the top of the ranked gene list *L*. The *ES* was calculated by walking down the list *L*. In detail, at a given position *i* in the ranked gene list *L* = < *g*_1_, *g*_2_,… *g*_n_>, we evaluated the fraction of genes in the pathway (*F*_*InP*_) weighted by their *propagation scores* and the fraction of genes not in the pathway (*F*_*NotP*_) as follows:


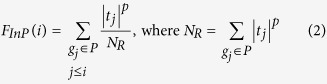



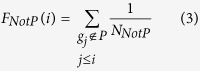


where *t*_*j*_ is the *propagation score* of gene *j, N*_*NotP*_ is the number of genes in list *L* not in the pathway, and *p* is used to weight the *propagation scores* of the genes in the pathway and was set to *p* = 1 in this study. With position *i* walking down the list *L*, a running-sum statistic *F*_*InP*_−*F*_*NotP*_ was calculated by increasing it when we encounter a gene in the pathway and decreasing it when we encounter genes not in the pathway. The *ES* of the pathway (*ES(P*)) was defined as follows:


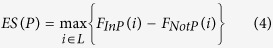


The *ES(P*) will be relatively high if the genes in the pathway are concentrated at the top of list *L*, but if the genes are randomly distributed in *L*, then the *ES(P*) will be correspondingly small.

We performed a permutation test to estimate the statistical significance (empirical *p-value*) of the *ES(P*). Specifically, we randomly selected a set of lncRNAs including the same number of lncRNAs with source nodes and mapped them to the CNC network. The *ES(P*) was then recomputed. A null distribution for the *ES* (designated as *ES*_NULL_) was generated after performing *N* permutations. The empirical *p-value* of the observed *ES* was calculated by comparing it with the set of scores in *ES*_NULL_, that is, *p-value* = *M/N*, where *M* is the number of *ES*_NULL_ values greater than the observed *ES(P*). In this study, for the examples, the number of permutations *N* was set at 1000. To correct for multiple comparisons, we adjusted the empirical *p-values* using the false discovery rate (FDR)^37^.

In fact, not all of the members of a pathway will be influenced by the combinatorial effects of the inputted lncRNAs. Therefore, it is meaningful to extract the core members of high-scoring pathways that contribute to the *ES(P*). Here, we defined the core genes of the pathway to be the genes in the pathway that appear in the ranked gene list *L* at or before the point where the *ES(P*) is obtained. The core genes of a pathway may account for the influence from the inputted lncRNAs.

## Results

### Identification of pathways influenced by dysregulated lncRNAs in prostate cancer

Our first case was a set of differentially expressed lncRNAs in prostate cancer. We downloaded RNA-Seq data on prostate cancer retrieved from the NCBI SRA database (SRA ID: SRP006731).These data sequenced the transcriptome of seven LNCaP prostate cancer cell lines and four normal prostate epithelial cell lines^38^. Raw RNA-Seq data were used to produce transcriptome assemblies by applying the “TopHat v2.0.13^23^+ Cufflinks v2.2.1^24^” pipeline with the default parameters. The expression values of lncRNAs and protein-coding genes were quantified using FPKM.

We applied Cuffdiff2^39^ to identify differentially expressed lncRNAs between the tumors and normal samples. An lncRNA was considered to be differentially expressed when the adjusted *p*-value of the lncRNA in Cuffdiff2 was less than 0.01. A total of 60 differentially expressed lncRNAs were identified in the CNC network (see Supplementary Table S3 for details). We subsequently inputted these lncRNAs into LncRNAs2Pathways to identify KEGG pathways. With an FDR < 0.01 pathway significance threshold, LncRNAs2Pathways yielded 20 statistically significant pathways (Table 1). Most of these pathways are readily interpreted in terms of the current knowledge of prostate cancer. For instance, the inhibition of oxidative phosphorylation may trigger the reactive oxygen species-mediated death of human prostate cancer cells^40^; chemokine receptors are associated with enhanced adhesive and invasive activities^41^; cell signaling and regulators of the cell cycle are proposed to be molecular targets for prostate cancer prevention^42^; the suppression of focal adhesion kinase activity is shown to precede the induction of apoptosis of prostate cancer cells^43^; gap junctional intercellular communication is decreased in prostate cancer^44^; and regulation of the actin cytoskeleton is proposed to be associated with cancer cell migration and invasion^45^.

**Figure 2:**
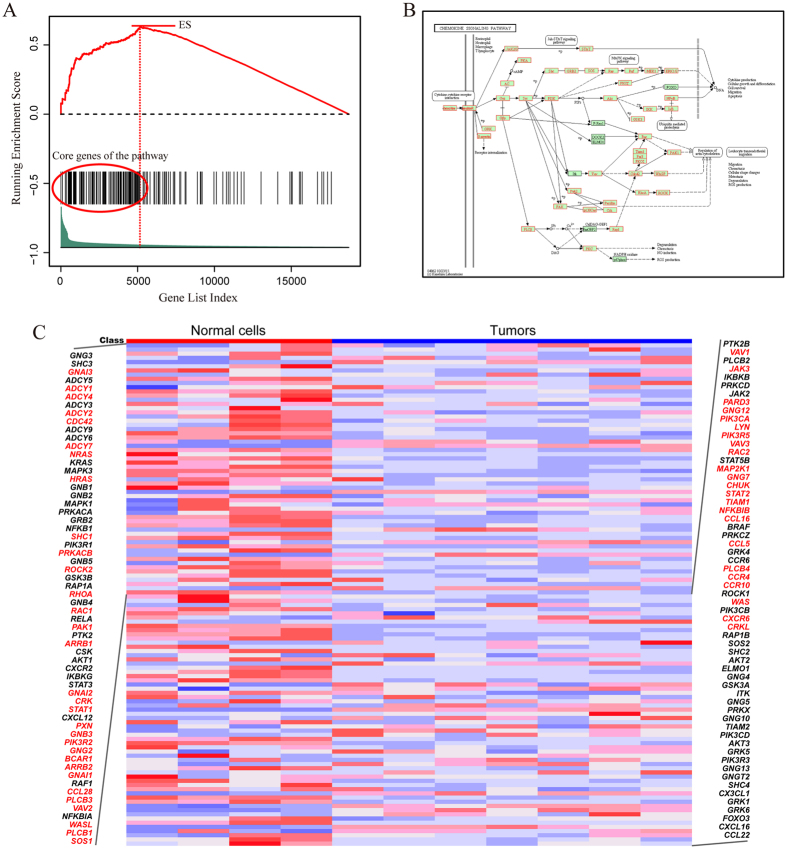
Running *ES* and annotating core protein-coding genes to the chemokine signaling pathway. (**A**) A running-sum statistic is calculated by walking down the protein-coding gene list, and the statistic’s maximum deviation from zero is used as the *ES(P*). (**B**) Chemokine signaling pathway in the KEGG database^34^,^35^. The gene products that correspond to the core protein-coding genes are annotated in red. (**C**) Heatmap of the expression levels of protein-coding genes in the pathway. Differentially expressed core protein-coding genes are marked in red.

We take the chemokine signaling pathway as an example to explain the rationale underlying LncRNAs2Pathways. Specifically, we mapped the differentially expressed lncRNAs to the CNC network and calculated the *propagation scores* of protein-coding genes using the RWR algorithm. A protein-coding gene list *L* was formed by ranking the genes according to their *propagation scores*, which reflect the degree of influence of each of the differentially expressed lncRNAs. The genes in the chemokine signaling pathway were mapped to the ranked list *L*, and 186 genes were obtained (Fig. 2A). The goal of LncRNAs2Pathways is to determine whether the genes in the pathway tend to occur toward the top of the ranked gene list *L*, in which case the pathway may be influenced by the combinatorial effects of differentially expressed lncRNAs. We calculated an *ES* that reflects the degree to which the pathway genes cluster toward the top of the ranked list *L*. A running-sum statistic was calculated by walking down the ranked list *L*, increasing it when we encounter a gene in the pathway and decreasing it when we encounter genes not in the pathway (Fig. 2A). The *ES* is the maximum value of the statistic. The protein-coding genes that contributed to the *ES* were defined as core genes of the pathway, and 131 core genes were obtained (Supplementary Table S4).

We then mapped the core genes to the pathway graph in KEGG database, and a number of gene products were annotated (Fig. 2B). Most of these genes have been reported to be associated with the progression of prostate cancer. Activated cell division cycle 42 (CDC42)-associated kinase Ack1 promotes prostate cancer progression^46^. Ras homolog family member A (RHOA) regulates clinically relevant androgen action in prostate cancer cells^47^. Protein kinase C, alpha (PRKCA) mediates epidermal growth factor receptor transactivation in human prostate cancer cells^48^. Interestingly, these protein-coding genes are located close to the differentially expressed lncRNAs in the CNC network. Specifically, the shortest distances from CDC42 and RHOA to LncRNA metastasis-associated lung adenocarcinoma transcript 1 (MALAT1) are all two, and the shortest distances from PRKCA to lncRNAs nuclear paraspeckle assembly transcript 1 (NEAT1), LINC00963 and MALAT1 are all three. More importantly, these lncRNAs are reported to be associated with the development of prostate cancer. MALAT1 down-regulation by siRNA inhibits prostate cancer cell growth, invasion and migration^49^; NEAT1 drives prostate cancer growth by altering the epigenetic landscape of target gene promoters to favor transcription^50^; and LINC00963 is involved in the transition of prostate cancer from androgen dependent to androgen independent^51^. These results indicate that the core genes in this pathway may be influenced by the dysregulated lncRNAs in the development of prostate cancer.

To further validate our results, we tested the expression changes of core genes in the significant pathways between prostate tumors and normal samples. We applied the same RNA-Seq data of prostate cancer and quantified the protein-coding gene expression levels with FPKM. The fold-change (FC) method was used to evaluate the changes in gene expression levels between tumors and normal samples. A coding gene was considered to be differentially expressed when the |log2 (FC)| value of the gene exceeded 1 (i.e., FC > 2 or FC < 0.5). For the chemokine signaling pathway, more than 40% (54/131) of its core genes are differentially expressed (Fig. 2C). This significant pathway was identified by applying the LncRNAs2Pathways method to the set of differentially expressed lncRNAs and observing that the expressions of a number of core genes in the pathways changed accordingly. Thus, the coding genes in the pathway may be influenced by the differentially expressed lncRNAs in the progression of prostate cancer. We also tested other significant pathways, such as cell cycle and gap junction pathways, and found that 43.6% (41/94) and 46.2% (30/65) of their core genes are differentially expressed in these pathways, respectively (Supplementary Figures S2 and S3).

### Identification of pathways influenced by the lncRNAs in glioma

Our second case was a set of lncRNAs associated with glioma, which was downloaded from Lnc2Cancer, a manually curated database of experimentally supported cancer-associated lncRNAs^52^. We obtained 11 glioma-associated lncRNAs in the CNC network (Supplementary Table S5). We inputted these lncRNAs to LncRNAs2Pathways, and 24 significant pathways (FDR < 0.01) were identified (Table 2). Most of these pathways are clearly related to glioma. For instance, the activation of the mitogen-activated protein kinase (MAPK) signaling pathway is associated with the chemosensitivity of glioma cells^53^; the neuregulin-1/ERBB receptor signaling cascade contributes to enhancing the survival of human astrocytic glioma cells^54^; impairment of the cell cycle is associated with the growth inhibition of human glioma cells^55^; blocking the focal adhesion pathway has the potential to be an efficacious treatment for human gliomas^56^; and neurotrophin signaling could be a target for the combinatorial treatment of malignant glioma^57^. The glioma pathway was also found to be significant. We took this pathway as an example to illustrate how the significant pathways were identified by LncRNAs2Pathways.

**Figure 3:**
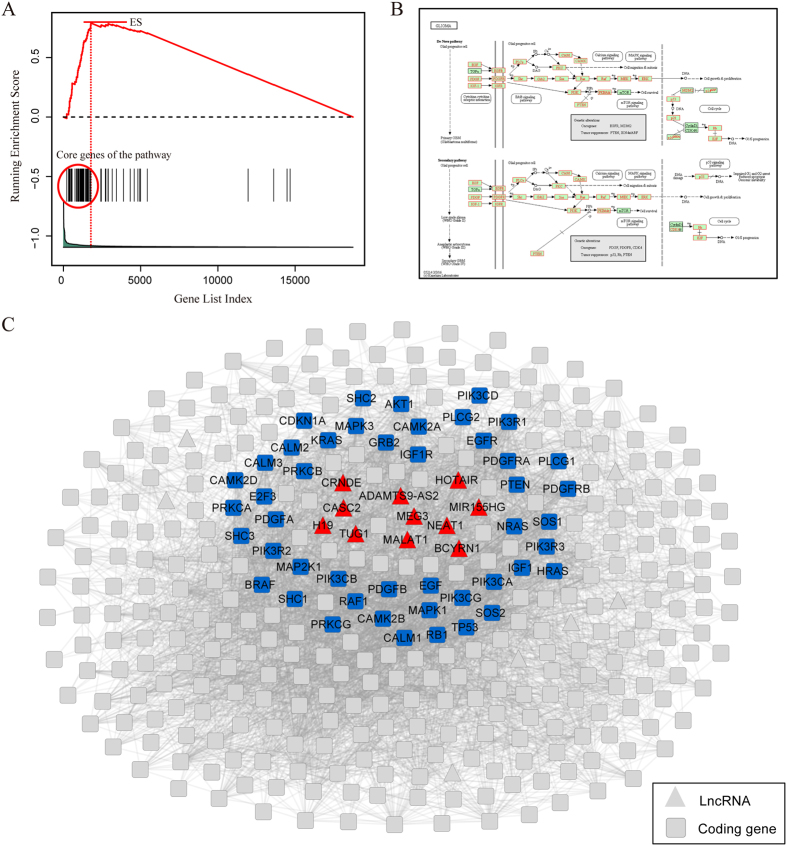
Running ES and annotating core protein-coding genes of the glioma pathway. (**A**) A running-sum statistic is calculated by walking down the protein-coding gene list, and the statistic’s maximum deviation from zero is used as the *ES(P*). (**B**) Glioma pathway in the KEGG database^34^,^35^. The gene products that correspond to the core protein-coding genes are annotated in red. (**C**) Connected sub-network of core protein-coding genes (marked with blue) and inputted lncRNAs (marked with red) in the CNC network.

The 11 glioma-associated lncRNAs were mapped to the CNC network. The *propagation scores* of the protein-coding genes, which reflect the degree of coding genes influenced by the inputted lncRNAs, were calculated by the RWR algorithm. A coding gene list was constructed by ranking the coding genes according to their *propagation scores*. The genes in the glioma pathway were mapped to the ranked coding gene list. If the coding genes in the pathway cluster at the top of the ranked list, the pathway will be regulated by the combinatorial effects of the lncRNA set. We calculated a running-sum statistic by walking down the list (Fig. 3A), and the maximum value of the statistic was used as the *ES(P*), which reflects the extent to which the pathway is overrepresented at the top of the ranked gene list. The core genes of the pathway that contribute to the *ES* were extracted and annotated on the original pathway graph (Fig. 3B). In the pathway, almost all the annotated genes were reported to be associated with the initiation and progression of glioma. Epidermal growth factor receptor (EGFR) promotes the malignant potential of glioma cells by interacting with the functional subunit of the cysteine/glutamate transporter xc-system (xCT) at the cell surface^58^; the expression of insulin-like growth factor 1 receptor (IGF1R) was found to be associated with the proliferation, migration, invasion, and tumorigenesis of glioma cells^59^. The phosphoinositol phosphatase activity of phosphatase and tensin homolog (PTEN) mediates serum-sensitive G1 growth arrest in glioma cells^60^. Neuroblastoma RAS viral oncogene homolog (NRAS) was identified as promoting oncogenesis in glioma stem cells^61^, and the upregulation of growth factor receptor bound protein 2 (GRB2)-associated binder 2 was demonstrated to be correlated with glioma^62^. Moreover, we extracted the connected sub-network among core protein-coding genes and inputted lncRNAs in the CNC network, which revealed that none of the core genes in the pathway were direct neighbors of the inputted lncRNAs. However, they were located close to the inputted lncRNAs (Fig. 3C). For instance, the average shortest distance from EGFR, IGF1R, PTEN, NRAS or GRB2 to the inputted lncRNAs did not exceed three. This result suggests that the core genes may be regulated by the lncRNAs. If we focus only on the co-expressed protein-coding genes of the lncRNAs, this pathway would be neglected. Our LncRNAs2Pathways method, which applies the global network propagation algorithm, successfully identified this pathway.

### Identification of pathways influenced by lncRNAs in pancreatic cancer

The third case we used was a set of lncRNAs associated with pancreatic cancer. We downloaded this set from the Lnc2Cancer database, and a total of nine pancreatic-cancer associated lncRNAs were identified in the CNC network (Supplementary Table S6). With FDR < 0.01, LncRNAs2Pathways obtained seven statistically significant pathways (Table 3), examples of which include the following: arachidonic acid metabolism, in which lipoxygenases, which are the key constituents, play a critical role in pancreatic cancer cell proliferation^63^; oxidative phosphorylation, whose KCa3.1 channel was identified as a regulator in a subset of pancreatic carcinoma cell lines^64^; the reduction of retinoids and their receptors, which is associated with pancreatic cancer patient survival^65^; and the overexpression of ribosomal proteins, which can promote tumorigenesis by interacting with the p53 tumor suppressor^66^. For the significant pathways, such as arachidonic acid metabolism, 14 core genes were identified (Supplementary Figure S4A). Because the core genes contributed to the *ES(P*), they might tend to be regulated by the pancreatic-cancer associated lncRNAs in the development of cancer. By mapping the core genes to the pathway graph (Supplementary Figure S4B), we found that 10 enzymes corresponding to the catalytic reaction of arachidonate were annotated. Interestingly, arachidonate has been reported to play a key role in carcinogenesis^67^. These results indicate that dysfunction of the arachidonic acid metabolism pathway may be regulated by the combinatorial effects of lncRNAs associated with pancreatic cancer.

### Comparing two studies of prostate cancer

**Figure 4:**
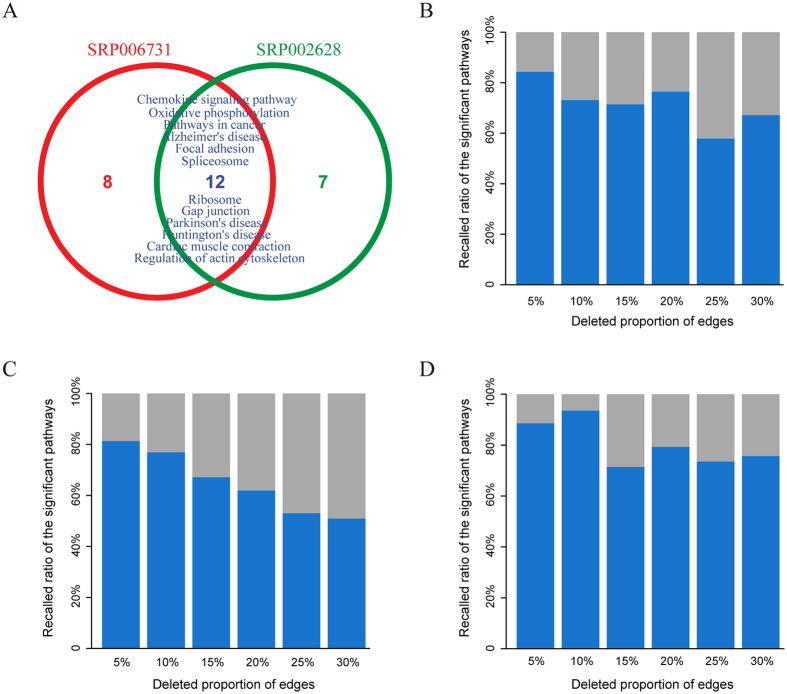
Performance of LncRNAs2Pathways. (**A**) Venn diagram of the overlapping significant pathways identified by two prostate cancer-associated lncRNA sets derived from the SRP006731 and SRP002628 datasets. (**B**–**D**) The mean recalled ratio of the significant pathways calculated by randomly deleting 5%, 10%, 15%, 20%, 25% and 30% of edges in the CNC network for the three lncRNA sets associated with prostate cancer, glioma and pancreatic cancer, respectively.

We applied LncRNAs2Pathways to two independent lncRNA sets associated with prostate cancer to test whether reproducible results could be obtained. The two lncRNA sets are as follows: (*i*) 60 differentially expressed lncRNAs (calculated by Cuffdiff2^39^, adjusted *p*-value < 0.01) derived from RNA-Seq data proposed by Kim *et al*. (SRA ID: SRP006731)^38^, which was used in our first case; (*ii*) 8 differentially expressed lncRNAs derived from RNA-Seq data proposed by Kannan *et al*. (SRA ID: SRP002628)^68^. Only one lncRNA was shared between the two lncRNA sets. Our goal was to examine whether LncRNAs2Pathways could identify consistent significant pathways. With FDR < 0.01, 20 and 19 pathways were identified by LncRNAs2Pathways in the two sets, respectively. We found that over 50% of the statistically significant pathways (12 pathways) were shared between the two studies (Fig. 4A). The shared pathways, such as the oxidative phosphorylation, chemokine signaling, focal adhesion, and gap junction pathways, have been proposed to be clearly related to the progression of prostate cancer^40^,^41^,^43^,^44^. To explain why the same significant pathways were identified using different lncRNA sets, we further compared the core genes in the pathways. For example, in the chemokine signaling pathway, 131 and 137 core genes were found in the two studies, and 121 were shared between them. The large overlap between the core genes in the two studies indicates that although the lncRNAs in the two sets are different, they may regulate consistent functional pathways associated with prostate cancer.

We then introduced a third prostate cancer-associated lncRNA set downloaded from the Lnc2Cancer database, which includes 28 lncRNAs. To provide a more general comparison, we compared significant pathways other than those that satisfied FDR < 0.01. Specifically, the top scoring 30 pathways determined by each of the three lncRNA sets were considered. Interestingly, half of these pathways (15/30) were shared across the three studies. The above analysis shows that LncRNAs2Pathways could obtain reproducible results across the three lncRNA sets associated with prostate cancer.

### Robustness analysis for the CNC network

The robustness analysis was performed by deleting a portion of the edges in the CNC network. Specifically, we randomly deleted 5%, 10%, 15%, 20%, 25% and 30% of the edges in the CNC network and repeated the LncRNAs2Pathways method 20 times for each deletion. We then calculated the mean recalled ratio of the original significant pathways for each deletion separately. We performed robustness analysis on the three aforementioned lncRNA sets of interest, namely, the differentially expressed lncRNA set in prostate cancer, glioma-associated lncRNA set and pancreatic cancer-associated lncRNA set. For the lncRNA set in prostate cancer, 20 statistically significant pathways were identified (FDR < 0.01). With 5%, 10%, 15%, and 20% of edges deleted, the mean recalled ratio of the significant pathways fell slowly, and the mean recalled ratio of the significant pathways exceeded 70%. With 25% and 30% edges deleted, the results were slightly inferior, but the mean recalled ratio of the significant pathways remained above 55% (Fig. 4B). We repeated this operation on the glioma-associated lncRNA set and pancreatic cancer-associated lncRNA set, obtaining similar results (Fig. 4C and D). The results of these experiments show that LncRNAs2Pathways is robust against edge deletion in the CNC network. This finding could be explained by the fact that the global network propagation algorithm used by our method can propagate the effects of lncRNAs effectively, even if the CNC network is incomplete.

### Comparison of LncRNAs2Pathways with other methods

To determine whether LncRNAs2Pathways can provide new insight into the identification of the pathways regulated by a given lncRNA set, we compared the results of LncRNAs2Pathways with those of two other methods: LncRNA2Function^18^ and Co-LncRNA^19^. We applied all three methods to identify significant pathways based on the differentially expressed lncRNAs in prostate cancer and the lncRNAs associated with glioma. For the differentially expressed lncRNAs in prostate cancer, a total of 38 statistically significant pathways were identified by the three methods using the default threshold for each method (Supplementary Table S7). Specifically, LncRNA2Function found 18 statistically significant pathways (Benjamini-Hochberg (BH)‘s FDR < 0.01), Co-LncRNA found 3 statistically significant pathways (Bonferroni adjusted *p*-value < 0.01), and LncRNAs2Pathways identified 20 significant pathways (BH’s FDR < 0.01). By comparing these results, we found that 17 pathways identified by LncRNAs2Pathways were missed by both other methods (Supplementary Table S7). Some of the missed pathways, such as the oxidative phosphorylation, cell cycle and focal adhesion pathways, have been well documented as being related to prostate cancer^40^,^42^,^43^. The pathways identified by LncRNAs2Pathways but missed by the other two methods may be attributed to the fact that LncRNA2Function and Co-LncRNA mainly use the hypergeometric test to identify significant pathways by annotating the protein-coding genes that are significantly co-expressed with the lncRNAs in the pathways. In contrast, LncRNAs2Pathways identifies significant pathways using a global network propagation algorithm. In LncRNAs2Pathways, the RWR algorithm was applied to calculate the *propagation score* for each protein-coding gene, which reflects the average network-proximity of the coding gene to the inputted lncRNAs. A coding gene located close to the lncRNAs will be assigned a larger *propagation score.* The Kolmogorov–Smirnov-like statistic weighted by the *propagation score* was used to evaluate the combinatorial effect of the lncRNAs in the biological pathways. Thus, LncRNAs2Pathways could identify new pathways whose genes are not co-expressed with the inputted lncRNAs but are close to the lncRNAs. We also applied the three methods to the set of lncRNAs associated with glioma, and a total of 37 statistically significant pathways were identified (Supplementary Table S8). Interestingly, LncRNAs2Pathways exclusively identified 20 significant pathways. The above results indicate that LncRNAs2Pathways may uncover some new biological pathways associated with a given lncRNA set and may therefore complement currently used methods.

## Discussion

LncRNAs have been reported to be involved in a wide range of biological processes^1^,^2^,^3^,^4^,^5^ and complex human diseases^6^,^7^,^8^. From a systems biology perspective, the LncRNAs generally perform their biological functions jointly rather than individually^16^,^17^. Thus, the identification of biological functions influenced by the combinatorial effects of a set of lncRNAs of interest is indispensable. Our study is the first to predict probable functions regulated by the combinatorial effects of a set of lncRNAs of interest based on a global network propagation strategy. In this study, we first constructed a CNC network by integrating RNA-Seq datasets and protein–protein interactions. Then, a set of lncRNAs was mapped to the network as source nodes, and the RWR algorithm was applied to evaluate the extent of protein-coding genes influenced by the combinatorial effects of the source nodes. Finally, we used a Kolmogorov–Smirnov-like statistic weighted by the influenced extent to prioritize the functional pathways. The results show that LncRNAs2Pathways can effectively identify the functional pathways associated with the lncRNA sets. To make the method more broadly applicable, we have implemented LncRNAs2Pathways as an R-based tool, which is freely available on the Comprehensive R Archive Network (CRAN; https://cran.r-project.org/web/packages/LncPath/). The users input an interesting lncRNA set, and the significant pathways are then returned. It is expected that LncRNAs2Pathways could facilitate the study of lncRNAs and further guide experimental design for biologists.

We collected 28 human RNA-Seq datasets under different biological conditions. For each dataset, we extracted the co-expression relationships among genes (both lncRNAs and protein-coding genes) through a strict computational pipeline (see the Methods section). The co-expressed gene pairs with the same correlation direction (i.e., positive or negative) in more than a certain number of datasets were retained for further analysis. To determine the number of datasets, we analyzed several network parameters with different dataset number cutoffs (from two to nine) (Supplementary Table S9). The size of the network naturally decreased as the cutoff value increased. The co-expression networks were confirmed with cutoffs of three or more datasets whose degree distributions approximately obey a power law distribution (Supplementary Figure S5), as observed for many biological networks and other types of networks^69^,^70^. Other studies of co-expression among genes also consider two genes to be co-expressed based on at least three datasets^11^,^71^. In this study, based on the size and quality of the networks, we only retained co-expressed gene pairs with a cutoff of three datasets for further analysis. Alternatively, the co-expression relationships among genes can also be extracted by combining *p*-values or effect sizes of Pcc across multiple datasets^72^. Because our method is flexible, researchers could also apply the algorithm to their network of interest using our software package.

In our study, to model real biological processes as accurately as possible, we further integrated gene co-expression relationships with protein–protein interactions to construct the CNC network. The CNC network consists of 28,613 nodes (11,391 lncRNAs and 17,222 protein-coding genes) and 295,698 edges (see Supplementary Table S2 for details about the CNC network). Previously, the integration of gene co-expression relationships with protein–protein interactions was performed by Guo *et al*.^12^ to predict probable functions for lncRNAs individually; however, we used this strategy to identify the function pathways influenced by the combinatorial effects of a set of lncRNAs. Moreover, the method used by Guo *et al*. used re-annotated microarray data corresponding to only 1,713 lncRNAs to extract gene co-expression relationships; in contrast, the CNC network covers 11,391 lncRNAs.

To investigate the influence of the CNC network, we performed data removal tests by randomly deleting 5%, 10%, 15%, 20%, 25% and 30% of edges in the CNC network. For each deletion, the LncRNAs2Pathways method was repeated 20 times. For the lncRNA set that was differentially expressed in prostate cancer, the mean recalled ratio of the original significant pathways remained above 55%, even after the deletion of up to 30% of edges in the CNC network (Fig. 4B). For the glioma-associated lncRNA set and pancreatic cancer-associated lncRNA set, we obtained similar results (Fig. 4C and D). These findings are attributable to the global network propagation algorithm, RWR^31^, used in our method. As long as the network is connected, this algorithm can propagate the functional effects of source nodes (i.e., the lncRNA set of interest) on the CNC network iteratively, even when the network is not comprehensive. Kohler *et al*. used this algorithm to prioritize candidate disease genes by mapping known disease genes to the protein interaction network^32^. We applied this algorithm to evaluate the extent of protein-coding genes influenced by the given set of lncRNAs in the CNC network.

Our method was designed to identify the pathways influenced by the combinatorial effects of a set of lncRNAs of interest, such as differentially expressed lncRNAs in a disease, and thus, the pathways identified by our method may be specifically regulated by the dysregulated lncRNAs in the disease state. To explain the effectiveness of LncRNAs2Pathways, we applied the method to three separate sets of lncRNAs. For the lncRNAs associated with prostate cancer, LncRNAs2Pathways identified 20 statistically significant pathways (FDR < 0.01, Table 1). Most of these pathways have been reported to be associated with the progression of prostate cancer. Because the pathways were obtained by mapping the differentially expressed lncRNAs in prostate cancer to the CNC network, they may be regulated by the combinatorial effects of the set of lncRNAs. We took the chemokine signaling pathway as an example to validate our results by testing the expression changes of core genes in significant pathways between tumors and normal samples. Interestingly, we found that more than 40% (54/131) of the core genes are differentially expressed (Fig. 2C). The pathway was identified by the differentially expressed lncRNAs, and the expression levels of the core genes in the pathways changed accordingly. Therefore, the pathway may indeed be regulated by the differentially expressed lncRNAs. For the lncRNAs associated with glioma, 24 significant pathways were identified (FDR < 0.01, Table 2), and the glioma pathway was identified as significant. By detecting the distances between the core genes of the pathway and inputted lncRNAs in the CNC network, we found that the core genes were not direct neighbors of the inputted lncRNAs but were located near the inputted lncRNAs in the CNC network (Fig. 3C). These results suggested that if we focused only on the protein-coding genes that are significantly co-expressed with the lncRNAs, this pathway might be neglected. The pathway obtained by LncRNAs2Pathways may be ascribed to the Kolmogorov–Smirnov-like statistic weighted by the *propagation score* used in our method.

Moreover, LncRNAs2Pathways can also prioritize the pathways influenced by a single lncRNA. For example, maternally expressed 3 (MEG3) is a maternally expressed, imprinted lncRNA gene that acts as a growth suppressor in tumor cells^73^. We inputted this lncRNA into LncRNAs2Pathways and obtained five statistically significant pathways with FDR < 0.01: (*i*) ribosome, (*ii*) focal adhesion, (*iii*) extracellular matrix (ECM)-receptor interaction, (*iv*) regulation of actin cytoskeleton, and (*v*) pathways in cancer. Interestingly, the changes in the ribosome biogenesis pathway reflect an increased incidence of tumor onset^74^; focal-adhesion kinase mediates cell proliferation, cell survival and cell migration^75^; the ECM-receptor interaction contributes to the venous metastases of hepatocellular carcinoma^76^; and regulation of the actin cytoskeleton is associated with cancer cell migration and invasion^45^. These results indicate that the significant pathways may be regulated by MEG3 in the beginning and during the progression of cancer.

Although a global network propagation strategy was successfully exploited in LncRNAs2Pathways for the functional annotation of a set of lncRNAs, our method can be improved in the following two ways. First, the cancer-associated lncRNA sets used in the study may be incomplete. In this work, we collected the lncRNAs associated with glioma and pancreatic cancers from the Lnc2Cancer database, and obtained 11 and 9 lncRNAs, respectively (Supplementary Tables S5 and S6). Although this database collected all experimentally supported cancer-associated lncRNAs from recent studies, a number of lncRNAs associated with the cancers remain to be validated. For prostate cancer, with an adjusted *p*-value < 0.01 in Cuffdiff2^39^, 60 differentially expressed lncRNAs were identified, but several moderately cancer-associated lncRNAs may have been missed. If the data on cancer-associated lncRNAs are improved, the specificity of LncRNAs2Pathways will be enhanced. Second, our method regarded the lncRNAs in the set of interest as equally important and did not consider the extent of the association between lncRNAs and cancer. Other network-based prediction algorithms should be incorporated to achieve better performance. Taken together, LncRNAs2Pathways not only provides a function predictor for lncRNA sets but also an open computational framework for the study of the combinatorial effects of lncRNAs.

## Additional Information

**How to cite this article:** Han, J. *et al*. LncRNAs2Pathways: Identifying the pathways influenced by a set of lncRNAs of interest based on a global network propagation method. *Sci. Rep.* **7**, 46566; doi: 10.1038/srep46566 (2017).

**Publisher's note:** Springer Nature remains neutral with regard to jurisdictional claims in published maps and institutional affiliations.

## Supplementary Material

Supplement Information

## Figures and Tables

**Table 1:** Pathways identified by LncRNAs2Pathways with FDR < 0.01 for the set of differentially expressed lncRNAs in prostate cancer.

Pathways	Size^a^	ES	P-value	FDR
Oxidative phosphorylation	114	0.81	<0.001	<0.001
Ribosome	87	0.87	<0.001	<0.001
Proteasome	44	0.82	<0.001	<0.001
Chemokine signaling pathway	186	0.63	<0.001	<0.001
Cell cycle	122	0.66	<0.001	<0.001
Focal adhesion	198	0.60	<0.001	<0.001
Gap junction	89	0.76	<0.001	<0.001
Regulation of actin cytoskeleton	211	0.60	<0.001	<0.001
Melanogenesis	101	0.63	<0.001	<0.001
Alzheimer’s disease	154	0.73	<0.001	<0.001
Parkinson’s disease	110	0.80	<0.001	<0.001
Huntington’s disease	168	0.74	<0.001	<0.001
Vibrio cholerae infection	54	0.78	<0.001	<0.001
Epithelial cell signaling in Helicobacter pylori infection	67	0.71	<0.001	<0.001
Pathogenic Escherichia coli infection	55	0.73	<0.001	<0.001
Pathways in cancer	324	0.55	<0.001	<0.001
Spliceosome	124	0.63	0.001	0.009
Cardiac muscle contraction	71	0.67	0.001	0.009
GnRH signaling pathway	98	0.63	0.001	0.009
Vasopressin-regulated water reabsorption	44	0.69	0.001	0.009

^a^Number of pathway genes in the CNC network.

**Table 2:** Pathways identified by LncRNAs2Pathways with FDR < 0.01 for the set of lncRNAs associated with glioma.

Pathways	Size^a^	ES	P-value	FDR
Ribosome	87	0.84	<0.001	<0.001
MAPK signaling pathway	262	0.65	<0.001	<0.001
ErbB signaling pathway	87	0.79	<0.001	<0.001
Cell cycle	122	0.74	<0.001	<0.001
Oocyte meiosis	109	0.73	<0.001	<0.001
Focal adhesion	198	0.72	<0.001	<0.001
Long-term potentiation	69	0.76	<0.001	<0.001
Neurotrophin signaling pathway	126	0.76	<0.001	<0.001
Regulation of actin cytoskeleton	211	0.71	<0.001	<0.001
Insulin signaling pathway	136	0.71	<0.001	<0.001
Huntington’s disease	168	0.70	<0.001	<0.001
Pathways in cancer	324	0.69	<0.001	<0.001
Glioma	65	0.79	<0.001	<0.001
Prostate cancer	89	0.78	<0.001	<0.001
Melanoma	71	0.78	<0.001	<0.001
Chronic myeloid leukemia	73	0.77	<0.001	<0.001
Chemokine signaling pathway	186	0.67	0.001	0.007
Adherens junction	73	0.75	0.001	0.007
T cell receptor signaling pathway	107	0.74	0.001	0.007
B cell receptor signaling pathway	74	0.75	0.001	0.007
Fc epsilon RI signaling pathway	76	0.77	0.001	0.007
GnRH signaling pathway	98	0.70	0.001	0.007
Alzheimer’s disease	154	0.71	0.001	0.007
Endometrial cancer	52	0.78	0.001	0.007

^a^Number of pathway genes in the CNC network.

**Table 3:** Pathways identified by LncRNAs2Pathways with FDR < 0.01 for the set of lncRNAs associated with pancreatic cancer.

Pathways	Size^a^	ES	P-value	FDR
Oxidative phosphorylation	114	0.81	<0.001	<0.001
Arachidonic acid metabolism	53	0.81	<0.001	<0.001
Retinol metabolism	53	0.82	<0.001	<0.001
Ribosome	87	0.93	<0.001	<0.001
Complement and coagulation cascades	69	0.88	<0.001	<0.001
Parkinson’s disease	110	0.80	<0.001	<0.001
Huntington’s disease	168	0.74	<0.001	<0.001

^a^Number of pathway genes in the CNC network.
