# Supplementary material for: LncRNAs2Pathways: Identifying the pathways influenced by a set of lncRNAs of interest based on a global network propagation method
Source: Sci Rep. 2017 Apr 20;7:46566. doi: 10.1038/srep46566 (PMC5397852; doi:10.1038/srep46566)
Supplement: Supplement Information [file srep46566-s1.doc]

**Supplementary Information**

**LncRNAs2Pathways: Identifying the pathways influenced by a set of lncRNAs of interest based on a global network propagation method**

Junwei Han^1,†^, Siyao Liu^1,†^, Zeguo Sun^1^, Yunpeng Zhang^1^, Fan Zhang^1^, Chunlong Zhang^1^, Desi Shang^1^, Haixiu Yang^1^, Fei Su^1^, Yanjun Xu^1^, Chunquan Li^3^,*, Huan Ren^2^,* and Xia Li^1^,*

^1^ College of Bioinformatics Science and Technology, Harbin Medical University, Harbin, 150081, PR China

^2^ Department of immunology, Harbin Medical University, Harbin 150081, PR China

^3^ School of Medical Informatics, Daqing Campus, Harbin Medical University, Harbin, 150081, PR China

*Correspondence and requests for materials should be addressed to C.L.( lcqbio@163.com), H.R.( huanren2009@126.com) or X.L.( lixia@hrbmu.edu.cn)

^†^These authors contributed equally to this work.

**Inventory of Supplementary Information**

1. Supplementary Figure S1

2. Supplementary Figure S2

3. Supplementary Figure S3

4. Supplementary Figure S4

5. Supplementary Figure S5

6. Supplementary Table S1

7. Supplementary Table S2

8. Supplementary Table S3

9. Supplementary Table S4

10. Supplementary Table S5

11. Supplementary Table S6

12. Supplementary Table S7

13. Supplementary Table S8

14. Supplementary Table S9


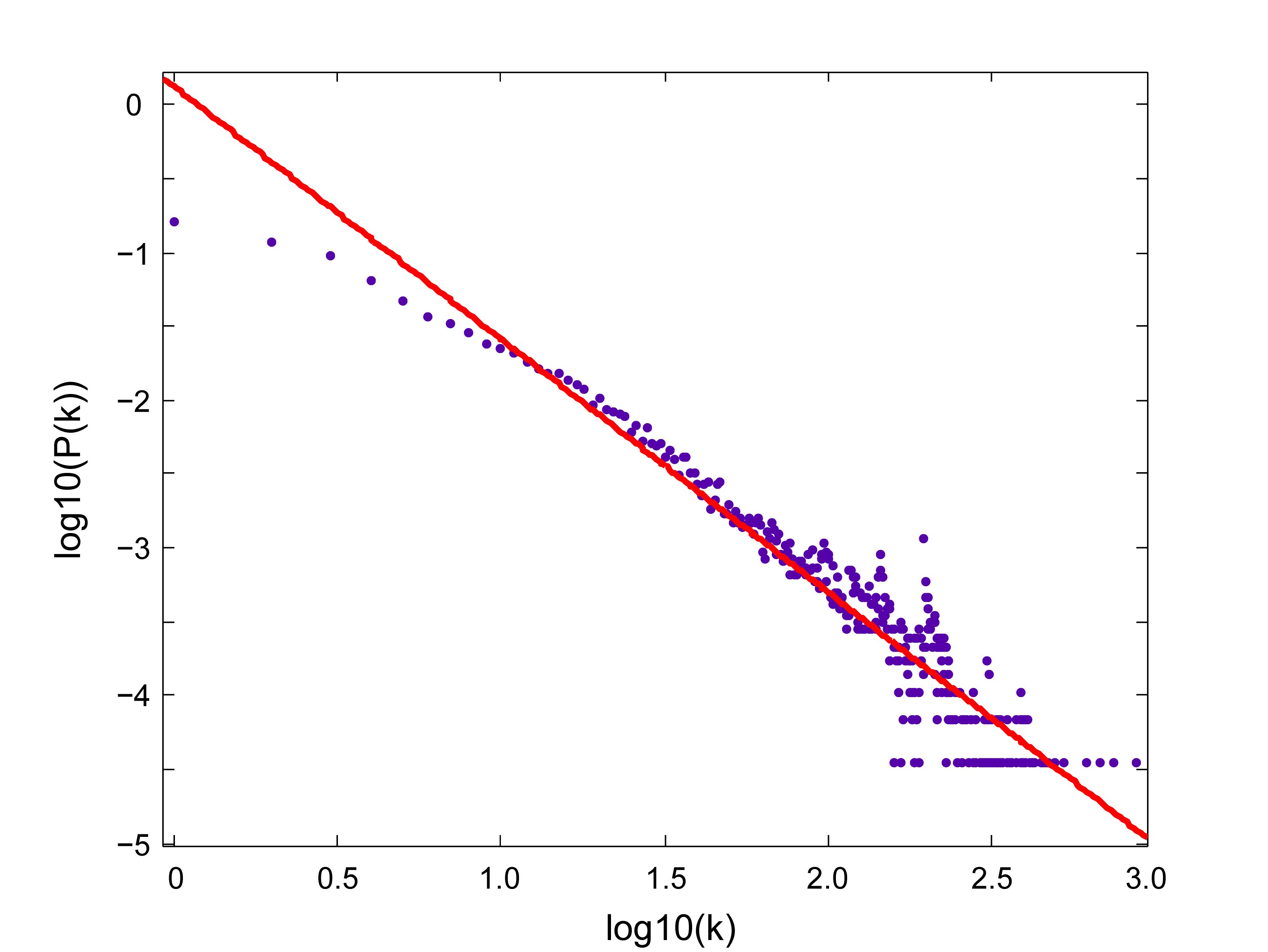


**Supplementary Figure S1.** The degree distribution of the of the coding–non-coding gene correlation. Here, k is degree, P(k) denotes the probability with a degree k.


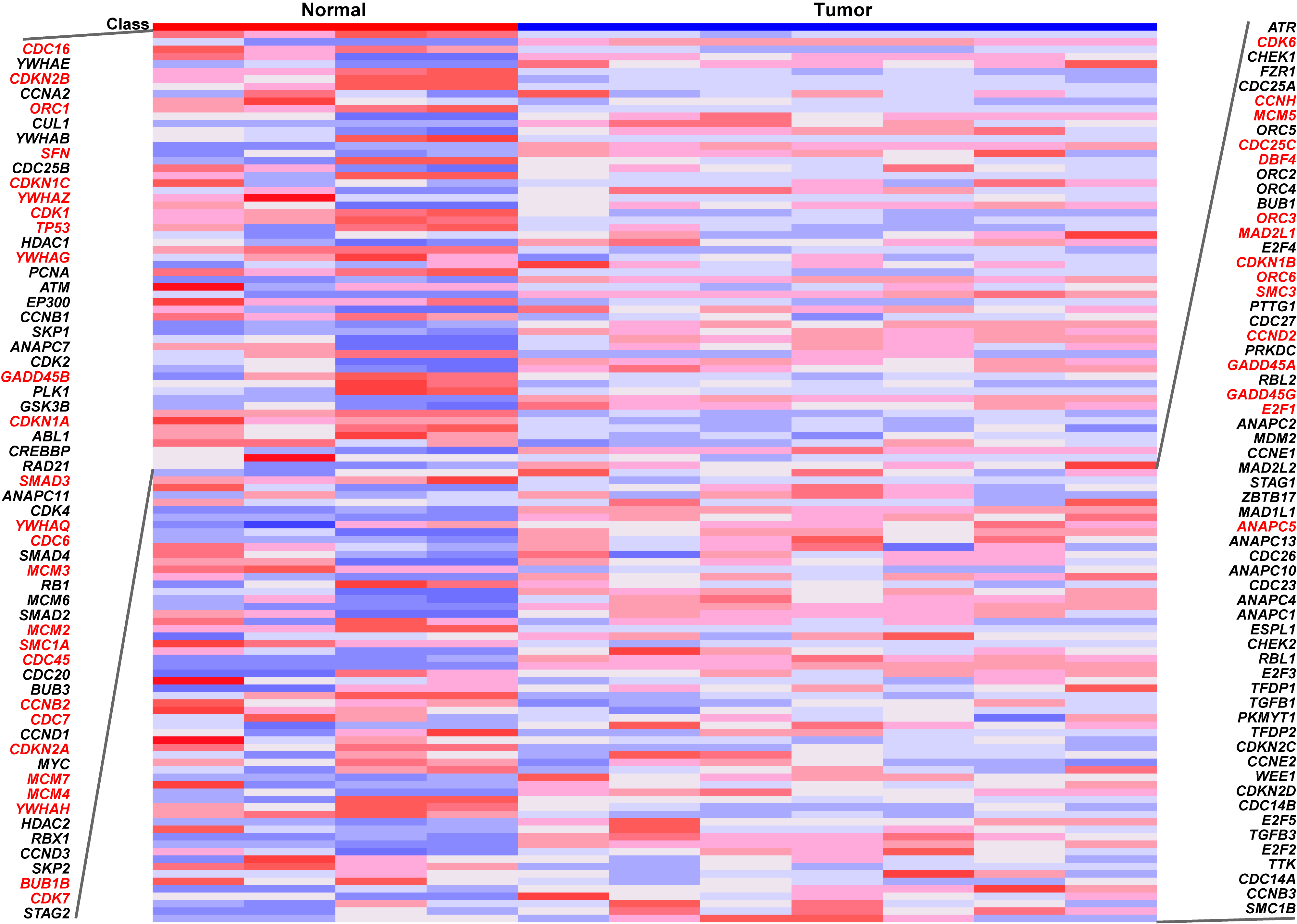


**Supplementary Figure S2.** Heatmap of the expression levels of protein-coding genes in the cell cycle pathway. Differently expressed core protein-coding genes are marked in red.


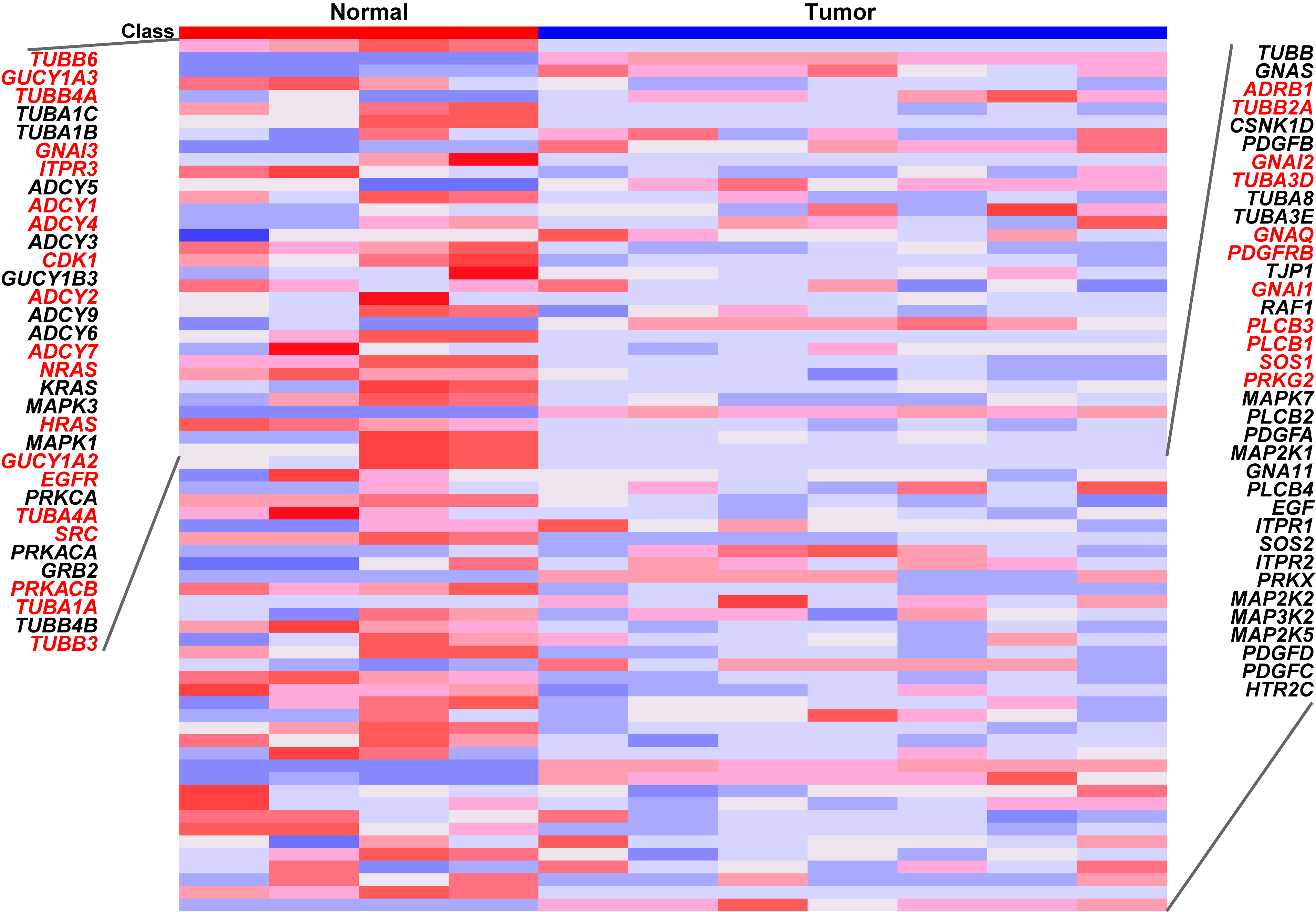


**Supplementary Figure S3.** Heatmap of the expression levels of protein-coding genes in the gap junction pathway. Differently expressed core protein-coding genes are marked in red.


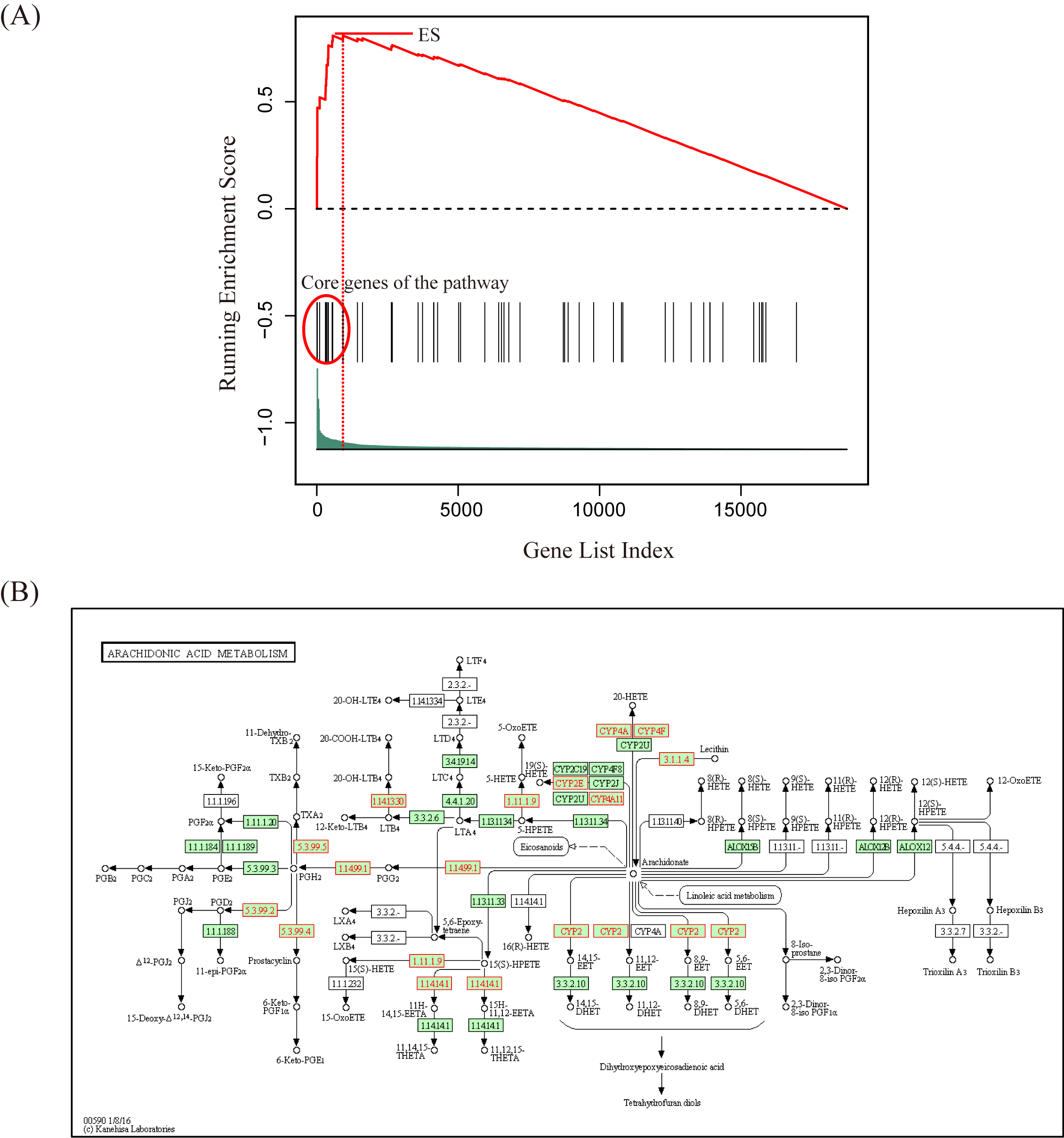


**Supplementary Figure S4.** Running enrichment score and annotating core protein-coding genes to the arachidonic acid metabolism pathway. (A) Running-sum statistic is calculated by walking down the protein-coding gene list, and the maximum deviation from zero of the statistic is used as the enrichment score of the pathway. (B) Arachidonic acid metabolism pathway in KEGG database^1,2^. The gene products that correspond to the core protein-coding genes are annotated in red.


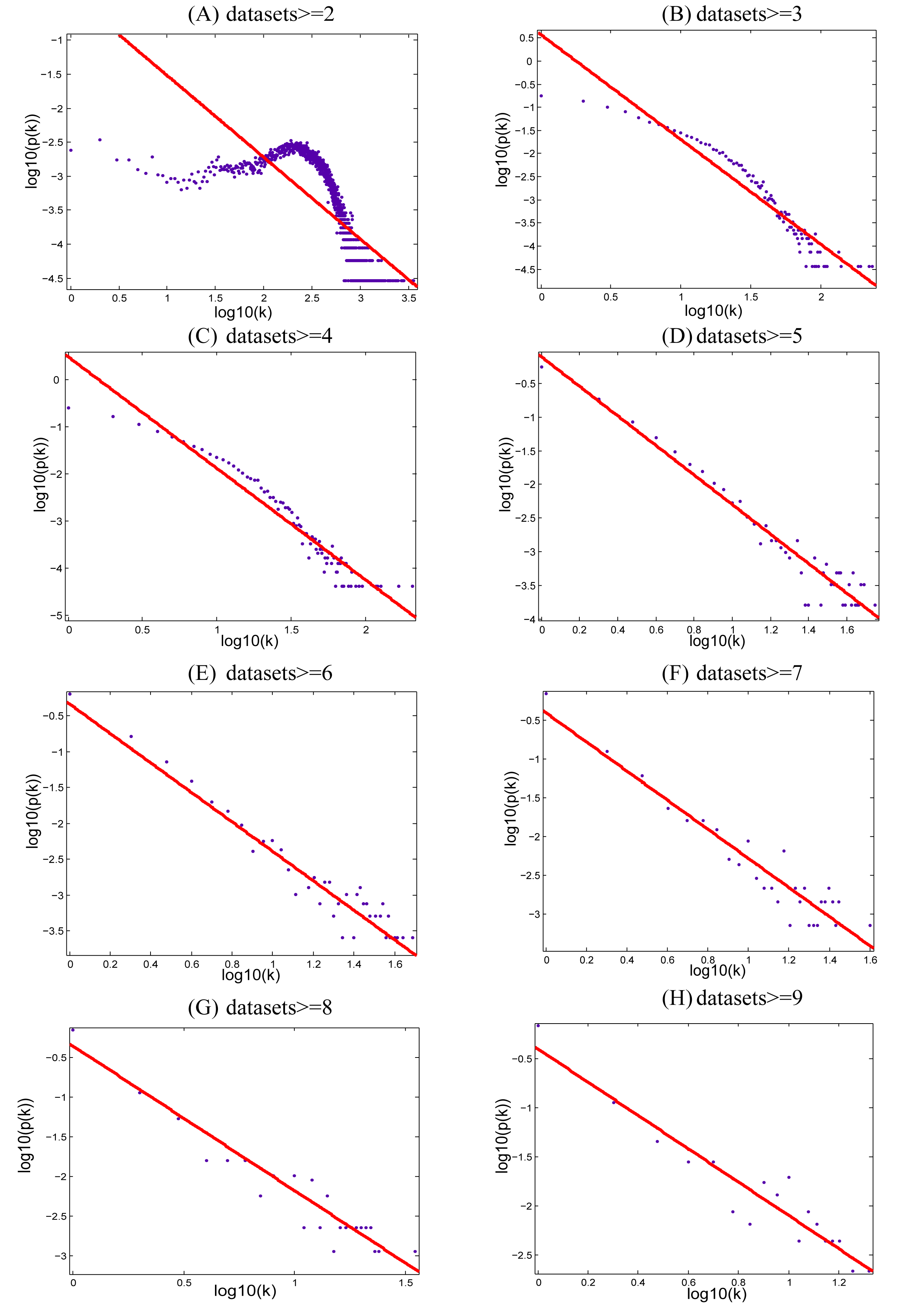


**Supplementary Figure S5. (A)-(H)** the degree distribution of the co-expressed networks confirmed by the cutoffs of dataset number from two to nine. Here, *k* is degree, *P(k)* denotes the probability with a degree *k*.

**Supplementary Table S1. Details for** **28 human** **RNA-Seq** **datasets**.

| **SRA ID** | **#Samples** | **Detail of Experiment Design** |
| --- | --- | --- |
| SRP000302 | 6 | Alternative Isoform Regulation in Human TissuTranscriptomes |
| SRP004879 | 6 | Whole transcriptome sequencing reveals gene expression and splicing differences in brain regions affected by Alzheimer's disease |
| SRP000626 | 6 | Deep surveying of alternative splicing complexity in the human transcriptome by high-throughput sequencing |
| SRP001119 | 6 | Developmental changes in human neocortical transcriptome revealed by RNA-seq |
| SRP003767 | 7 | Analysis of Cellular Transcript Abundance in 293T Cells Expressing SOX or muSOX |
| SRP003611 | 8 | Deep transcriptional sequencing analysis of human prostate adenocarcinoma and reference samples |
| SRP006676 | 8 | mRNA-seq of Human Airway Epithelial Cells |
| SRP004903 | 8 | A human transcriptome array for high-throughput clinical studies (with a comparison to RNA-seq technology) |
| SRP007494 | 9 | Integrative Annotation of Human Large Intergenic Non-Coding RNAs Reveals Global Properties and Specific Subclasses |
| SRP010483 | 10 | The human pancreatic islet transcriptome: impact of pro-inflammatory cytokines |
| ERP000573 | 10 | RNA and chromatin structure |
| ERP000418 | 10 | Gene expression profiles between normal and breast tumor genomes |
| SRP005411 | 11 | RNA-Seq Quantification of the Complete Transcriptome of Genes Expressed in the Small Airway Epithelium of Nonsmokers and Smokers |
| SRP006731 | 11 | RNA-Seq anlalysis of prostate cancer cell lines using Next Generation Sequencing |
| SRP013224 | 11 | Next-generation sequencing reveals HIV-1-mediated suppression of T cell activation and RNA processing and the regulation of non-coding RNA expression in a CD4+ T cell line |
| ERP000710 | 12 | Transciptome profiling of ovarian cancer cell lines |
| SRP007338 | 16 | Widespread regulated alternative splicing of single codons accelerates proteome evolution |
| SRP010166 | 16 | Deep Sequence Analysis of non-small cell lung cancer: Integrated analysis of gene expression, alternative splicing, and single nucleotide variations in lung adenocarcinomas with and without oncogenic KRAS mutations |
| SRP000727 | 16 | Alternative Isoform Regulation in Human Tissue Transcriptomes |
| ERP000992 | 18 | The effect of estrogen and progesterone and their antagonists in Ishikawa cell line compared to MCF7 and T47D cells |
| SRP005242 | 19 | A Comparison of Single Molecule and Amplification Based Sequencing of Cancer Transcriptomes: RNA-Seq Comparison |
| SRP002079 | 20 | Dynamic transcriptomes during neural differentiation of human embryonic stem cells |
| ERP000550 | 29 | Complete transcriptomic landscape of prostate cancer in the Chinese population using RNA-seq |
| SRP002628 | 30 | Comparative transcriptomic analysis of prostate cancer and matched normal tissue using RNA-seq |
| SRP010280 | 31 | Integrative genome-wide analysis reveals cooperative regulation of alternative splicing by hnRNP proteins |
| SRP005408 | 31 | Gene expression profile in postmortem hippocampus using RNAseq for addicted human samples |
| SRP005169 | 41 | Widespread splicing changes in human brain development and aging |
| ERP000546 | 48 | Illumina bodyMap2 transcriptome |

**Supplementary Table S2.** Details for the relationships in the CNC network.

| **Data source** | **Number of Genes** | **Number of Edges** |
| --- | --- | --- |
| HPRD | 9 617 protein-coding genes | 39 240 protein interactions |
| DIP | 1 240 protein-coding genes | 1 564 protein interactions |
| MINT | 5 547 protein-coding genes | 16 763 protein interactions |
| Reactome | 6 299 protein-coding genes | 139 797 protein interactions |
| Co-expression | 15 424 protein-coding genes  11 391 lncRNAs genes | 114 006 gene co-expressions  (88 028 coding–coding, 11 453 lncRNA–lncRNA and 18 055 coding–lncRNA) |
| Total | 17 222 protein-coding genes  11 391 lncRNAs genes | 181 692 protein interactions  104 391 gene co-expressions  9 615 both gene co-expressed and protein–protein interaction |

**Supplementary Table S3.** 60 differentially expressed lncRNAs between prostate cancer and normal samples in the CNC network.

| **Ensemble Gene ID** | **Gene Name** | **Adjusted p-value** |
| --- | --- | --- |
| ENSG00000203875 | SNHG5 | 0.000976826 |
| ENSG00000204054 | RP11-492E3.1 | 0.000976826 |
| ENSG00000230844 | ZNF674-AS1 | 0.000976826 |
| ENSG00000232164 | AC092669.3 | 0.000976826 |
| ENSG00000225255 | LA16c-83F12.6 | 0.000976826 |
| ENSG00000233864 | TTTY15 | 0.000976826 |
| ENSG00000235091 | WI2-85898F10.1 | 0.000976826 |
| ENSG00000244541 | RP11-167H9.6 | 0.000976826 |
| ENSG00000245532 | NEAT1 | 0.000976826 |
| ENSG00000247556 | OIP5-AS1 | 0.000976826 |
| ENSG00000251143 | RP11-849H4.4 | 0.000976826 |
| ENSG00000253369 | RP11-1081M5.1 | 0.000976826 |
| ENSG00000253553 | RP11-586K2.1 | 0.000976826 |
| ENSG00000255418 | RP11-266A24.1 | 0.000976826 |
| ENSG00000258232 | RP11-161H23.5 | 0.000976826 |
| ENSG00000258727 | RP11-66N24.3 | 0.000976826 |
| ENSG00000258301 | RP11-488C13.5 | 0.000976826 |
| ENSG00000259583 | RP11-66B24.4 | 0.000976826 |
| ENSG00000259380 | RP11-346D14.1 | 0.000976826 |
| ENSG00000261068 | RP11-7K24.3 | 0.000976826 |
| ENSG00000260260 | RP11-304L19.5 | 0.000976826 |
| ENSG00000261716 | RP11-196G18.22 | 0.000976826 |
| ENSG00000260032 | LINC00657 | 0.000976826 |
| ENSG00000259869 | AL022344.7 | 0.000976826 |
| ENSG00000238045 | AC009133.14 | 0.000976826 |
| ENSG00000260261 | RP11-480A16.1 | 0.000976826 |
| ENSG00000186594 | MIR22HG | 0.000976826 |
| ENSG00000263244 | RP11-473I1.10 | 0.000976826 |
| ENSG00000226137 | BAIAP2-AS1 | 0.000976826 |
| ENSG00000266402 | RP11-329L6.1 | 0.000976826 |
| ENSG00000263424 | CTD-2541J13.2 | 0.000976826 |
| ENSG00000267519 | CTD-3252C9.4 | 0.000976826 |
| ENSG00000163597 | SNHG16 | 0.000976826 |
| ENSG00000232677 | LINC00665 | 0.000976826 |
| ENSG00000267100 | ILF3-AS1 | 0.000976826 |
| ENSG00000267625 | RP11-1094M14.14 | 0.000976826 |
| ENSG00000268191 | CTD-2396E7.10 | 0.000976826 |
| ENSG00000227195 | RP3-410C9.1 | 0.000976826 |
| ENSG00000228830 | RP4-781K5.2 | 0.00184116 |
| ENSG00000254254 | RP11-17A4.2 | 0.00184116 |
| ENSG00000251562 | MALAT1 | 0.00264114 |
| ENSG00000265401 | RP11-138I1.4 | 0.00264114 |
| ENSG00000233621 | RP11-422J8.1 | 0.00340306 |
| ENSG00000230733 | AC092171.4 | 0.00340306 |
| ENSG00000223482 | RP11-322M19.1 | 0.00340306 |
| ENSG00000255198 | SNHG9 | 0.00340306 |
| ENSG00000263958 | RP11-676J15.1 | 0.00340306 |
| ENSG00000257764 | RP11-1143G9.4 | 0.00414482 |
| ENSG00000260244 | RP11-588K22.2 | 0.00480207 |
| ENSG00000248429 | RP11-597D13.9 | 0.00549182 |
| ENSG00000258441 | LINC00641 | 0.00549182 |
| ENSG00000232445 | RP11-132A1.4 | 0.00615487 |
| ENSG00000267321 | RP11-1094M14.11 | 0.00615487 |
| ENSG00000225470 | JPX | 0.00681492 |
| ENSG00000225339 | RP11-513I15.6 | 0.00681492 |
| ENSG00000257084 | U47924.27 | 0.00681492 |
| ENSG00000267062 | CTD-2659N19.10 | 0.00681492 |
| ENSG00000203499 | RP11-429J17.6 | 0.00747849 |
| ENSG00000261071 | RP1-223E5.4 | 0.00869375 |
| ENSG00000263585 | RP11-498C9.13 | 0.00869375 |

**Supplementary Table S4.** Detail information for each gene in the chemokine signaling pathway.

| **#** | **Gene** | **List location** | **Propagation score** | **Running-sum statistic** | **Core Gene** |
| --- | --- | --- | --- | --- | --- |
| 1 | GNG3 | 6 | 0.0423 | 0.0793 | YES |
| 2 | CCL20 | 100 | 0.0199 | 0.112 | YES |
| 3 | ADRBK1 | 285 | 0.0141 | 0.128 | YES |
| 4 | SHC3 | 288 | 0.0141 | 0.155 | YES |
| 5 | GNAI3 | 356 | 0.0122 | 0.174 | YES |
| 6 | ADCY5 | 480 | 0.00658 | 0.18 | YES |
| 7 | ADCY1 | 481 | 0.00654 | 0.192 | YES |
| 8 | ADCY4 | 506 | 0.0062 | 0.203 | YES |
| 9 | ADCY3 | 521 | 0.00596 | 0.213 | YES |
| 10 | ADCY2 | 550 | 0.00561 | 0.222 | YES |
| 11 | ADCY8 | 558 | 0.00557 | 0.232 | YES |
| 12 | CDC42 | 559 | 0.00556 | 0.243 | YES |
| 13 | ADCY9 | 560 | 0.00555 | 0.253 | YES |
| 14 | ADCY6 | 563 | 0.00555 | 0.263 | YES |
| 15 | ADCY7 | 564 | 0.00554 | 0.274 | YES |
| 16 | NRAS | 591 | 0.00533 | 0.283 | YES |
| 17 | KRAS | 635 | 0.00506 | 0.29 | YES |
| 18 | MAPK3 | 661 | 0.00493 | 0.298 | YES |
| 19 | HRAS | 668 | 0.0049 | 0.307 | YES |
| 20 | GNB1 | 680 | 0.00487 | 0.315 | YES |
| 21 | GNB2 | 694 | 0.00484 | 0.324 | YES |
| 22 | MAPK1 | 698 | 0.00483 | 0.333 | YES |
| 23 | PRKACA | 822 | 0.00447 | 0.334 | YES |
| 24 | GRB2 | 844 | 0.00441 | 0.341 | YES |
| 25 | NFKB1 | 850 | 0.00439 | 0.349 | YES |
| 26 | SHC1 | 856 | 0.00438 | 0.357 | YES |
| 27 | PIK3R1 | 877 | 0.00434 | 0.365 | YES |
| 28 | PRKACB | 898 | 0.0043 | 0.372 | YES |
| 29 | GNB5 | 928 | 0.00423 | 0.378 | YES |
| 30 | ROCK2 | 929 | 0.00423 | 0.386 | YES |
| 31 | GSK3B | 951 | 0.00419 | 0.393 | YES |
| 32 | RAP1A | 991 | 0.00412 | 0.398 | YES |
| 33 | RHOA | 998 | 0.0041 | 0.406 | YES |
| 34 | GNB4 | 1215 | 0.00378 | 0.401 | YES |
| 35 | CXCR4 | 1292 | 0.00368 | 0.404 | YES |
| 36 | RAC1 | 1302 | 0.00367 | 0.41 | YES |
| 37 | RELA | 1331 | 0.00362 | 0.416 | YES |
| 38 | PAK1 | 1366 | 0.00358 | 0.421 | YES |
| 39 | PTK2 | 1395 | 0.00355 | 0.426 | YES |
| 40 | ARRB1 | 1399 | 0.00355 | 0.432 | YES |
| 41 | CSK | 1470 | 0.00347 | 0.435 | YES |
| 42 | AKT1 | 1513 | 0.00343 | 0.439 | YES |
| 43 | CXCR2 | 1656 | 0.00333 | 0.438 | YES |
| 44 | IKBKG | 1822 | 0.00322 | 0.435 | YES |
| 45 | STAT3 | 1834 | 0.00321 | 0.441 | YES |
| 46 | PRKCB | 1910 | 0.00316 | 0.443 | YES |
| 47 | GNAI2 | 2018 | 0.00308 | 0.443 | YES |
| 48 | CRK | 2024 | 0.00308 | 0.448 | YES |
| 49 | GNG8 | 2047 | 0.00306 | 0.453 | YES |
| 50 | STAT1 | 2069 | 0.00305 | 0.457 | YES |
| 51 | FGR | 2077 | 0.00305 | 0.463 | YES |
| 52 | CXCL12 | 2118 | 0.00302 | 0.466 | YES |
| 53 | PXN | 2188 | 0.00296 | 0.468 | YES |
| 54 | GNB3 | 2300 | 0.0029 | 0.468 | YES |
| 55 | PIK3R2 | 2325 | 0.00289 | 0.472 | YES |
| 56 | GNG2 | 2418 | 0.00285 | 0.472 | YES |
| 57 | BCAR1 | 2421 | 0.00285 | 0.477 | YES |
| 58 | ARRB2 | 2541 | 0.00277 | 0.476 | YES |
| 59 | GNAI1 | 2545 | 0.00277 | 0.481 | YES |
| 60 | CXCL2 | 2552 | 0.00277 | 0.486 | YES |
| 61 | RAF1 | 2564 | 0.00276 | 0.491 | YES |
| 62 | CCL28 | 2729 | 0.00268 | 0.487 | YES |
| 63 | PLCB3 | 2776 | 0.00266 | 0.489 | YES |
| 64 | VAV2 | 2782 | 0.00265 | 0.494 | YES |
| 65 | NFKBIA | 2783 | 0.00265 | 0.499 | YES |
| 66 | WASL | 2822 | 0.00264 | 0.502 | YES |
| 67 | HCK | 2870 | 0.00262 | 0.504 | YES |
| 68 | PLCB1 | 2911 | 0.0026 | 0.507 | YES |
| 69 | SOS1 | 2918 | 0.00259 | 0.512 | YES |
| 70 | PTK2B | 2940 | 0.00258 | 0.515 | YES |
| 71 | VAV1 | 3136 | 0.0025 | 0.51 | YES |
| 72 | PLCB2 | 3144 | 0.00249 | 0.514 | YES |
| 73 | JAK3 | 3238 | 0.00246 | 0.514 | YES |
| 74 | IKBKB | 3258 | 0.00245 | 0.517 | YES |
| 75 | PRKCD | 3274 | 0.00244 | 0.521 | YES |
| 76 | CXCL1 | 3388 | 0.0024 | 0.519 | YES |
| 77 | JAK2 | 3443 | 0.00237 | 0.521 | YES |
| 78 | PARD3 | 3511 | 0.00234 | 0.522 | YES |
| 79 | CCR1 | 3588 | 0.00231 | 0.522 | YES |
| 80 | CCR5 | 3652 | 0.00228 | 0.523 | YES |
| 81 | GNG12 | 3728 | 0.00225 | 0.523 | YES |
| 82 | PIK3CA | 3737 | 0.00225 | 0.527 | YES |
| 83 | LYN | 3779 | 0.00223 | 0.529 | YES |
| 84 | PIK3CG | 3854 | 0.0022 | 0.529 | YES |
| 85 | PIK3R5 | 3892 | 0.00219 | 0.531 | YES |
| 86 | VAV3 | 3920 | 0.00218 | 0.534 | YES |
| 87 | CXCL10 | 3939 | 0.00217 | 0.537 | YES |
| 88 | RAC2 | 4045 | 0.00213 | 0.535 | YES |
| 89 | STAT5B | 4086 | 0.00211 | 0.537 | YES |
| 90 | MAP2K1 | 4111 | 0.00211 | 0.54 | YES |
| 91 | GNG7 | 4187 | 0.00208 | 0.54 | YES |
| 92 | CHUK | 4203 | 0.00207 | 0.543 | YES |
| 93 | STAT2 | 4206 | 0.00207 | 0.547 | YES |
| 94 | CCL23 | 4283 | 0.00204 | 0.546 | YES |
| 95 | TIAM1 | 4298 | 0.00203 | 0.549 | YES |
| 96 | NFKBIB | 4303 | 0.00203 | 0.553 | YES |
| 97 | CCL16 | 4366 | 0.00201 | 0.553 | YES |
| 98 | BRAF | 4428 | 0.00198 | 0.554 | YES |
| 99 | PRKCZ | 4435 | 0.00198 | 0.557 | YES |
| 100 | CCL5 | 4457 | 0.00198 | 0.56 | YES |
| 101 | GRK4 | 4475 | 0.00197 | 0.563 | YES |
| 102 | CXCL3 | 4497 | 0.00196 | 0.565 | YES |
| 103 | CCR6 | 4527 | 0.00195 | 0.567 | YES |
| 104 | CCL2 | 4555 | 0.00194 | 0.569 | YES |
| 105 | PPBP | 4589 | 0.00193 | 0.571 | YES |
| 106 | PLCB4 | 4666 | 0.0019 | 0.571 | YES |
| 107 | CCR4 | 4675 | 0.0019 | 0.574 | YES |
| 108 | CXCL5 | 4685 | 0.0019 | 0.577 | YES |
| 109 | CCL21 | 4700 | 0.00189 | 0.58 | YES |
| 110 | CCR10 | 4785 | 0.00185 | 0.579 | YES |
| 111 | ROCK1 | 4833 | 0.00184 | 0.58 | YES |
| 112 | CXCL13 | 4837 | 0.00184 | 0.583 | YES |
| 113 | WAS | 4850 | 0.00184 | 0.586 | YES |
| 114 | CCR2 | 4856 | 0.00184 | 0.589 | YES |
| 115 | PIK3CB | 4857 | 0.00184 | 0.593 | YES |
| 116 | CCR3 | 4884 | 0.00183 | 0.595 | YES |
| 117 | CXCR1 | 4887 | 0.00183 | 0.598 | YES |
| 118 | PF4 | 4938 | 0.00181 | 0.599 | YES |
| 119 | CCL19 | 4941 | 0.00181 | 0.602 | YES |
| 120 | CXCL9 | 4946 | 0.00181 | 0.605 | YES |
| 121 | CXCL6 | 4995 | 0.0018 | 0.606 | YES |
| 122 | CXCR3 | 5001 | 0.0018 | 0.609 | YES |
| 123 | CXCR6 | 5011 | 0.0018 | 0.612 | YES |
| 124 | CCR7 | 5029 | 0.00179 | 0.614 | YES |
| 125 | CXCL11 | 5053 | 0.00179 | 0.617 | YES |
| 126 | CXCR5 | 5054 | 0.00179 | 0.62 | YES |
| 127 | CCR8 | 5111 | 0.00177 | 0.62 | YES |
| 128 | CCL25 | 5127 | 0.00177 | 0.623 | YES |
| 129 | CRKL | 5140 | 0.00177 | 0.625 | YES |
| 130 | CCL27 | 5146 | 0.00177 | 0.628 | YES |
| 131 | CCR9 | 5150 | 0.00177 | 0.632 | YES |
| 132 | CCL11 | 5359 | 0.00172 | 0.624 | NO |
| 133 | RAP1B | 5366 | 0.00172 | 0.627 | NO |
| 134 | SOS2 | 5458 | 0.00169 | 0.625 | NO |
| 135 | SHC2 | 5555 | 0.00167 | 0.623 | NO |
| 136 | NCF1 | 5640 | 0.00165 | 0.621 | NO |
| 137 | AKT2 | 5805 | 0.0016 | 0.616 | NO |
| 138 | ELMO1 | 6061 | 0.00152 | 0.605 | NO |
| 139 | GNG4 | 6085 | 0.00152 | 0.606 | NO |
| 140 | GSK3A | 6093 | 0.00151 | 0.609 | NO |
| 141 | ITK | 6202 | 0.00149 | 0.606 | NO |
| 142 | GNG5 | 6334 | 0.00145 | 0.601 | NO |
| 143 | PRKX | 6379 | 0.00144 | 0.602 | NO |
| 144 | GNG10 | 6443 | 0.00143 | 0.601 | NO |
| 145 | GNGT1 | 6640 | 0.00138 | 0.593 | NO |
| 146 | TIAM2 | 6742 | 0.00135 | 0.59 | NO |
| 147 | PIK3CD | 6764 | 0.00134 | 0.592 | NO |
| 148 | AKT3 | 6842 | 0.00132 | 0.59 | NO |
| 149 | GRK5 | 6872 | 0.00132 | 0.591 | NO |
| 150 | PIK3R3 | 6999 | 0.00129 | 0.587 | NO |
| 151 | GNG13 | 7128 | 0.00127 | 0.582 | NO |
| 152 | XCL1 | 7324 | 0.00123 | 0.574 | NO |
| 153 | XCR1 | 7396 | 0.00122 | 0.572 | NO |
| 154 | XCL2 | 7419 | 0.00122 | 0.573 | NO |
| 155 | CCL18 | 7737 | 0.00114 | 0.558 | NO |
| 156 | RASGRP2 | 7807 | 0.00113 | 0.557 | NO |
| 157 | PRKACG | 8088 | 0.00108 | 0.544 | NO |
| 158 | GNGT2 | 8149 | 0.00107 | 0.543 | NO |
| 159 | SHC4 | 8886 | 0.000946 | 0.505 | NO |
| 160 | CX3CL1 | 9078 | 0.000921 | 0.496 | NO |
| 161 | GRK1 | 9124 | 0.000915 | 0.495 | NO |
| 162 | GNG11 | 9195 | 0.000906 | 0.493 | NO |
| 163 | GRK6 | 9965 | 0.000805 | 0.453 | NO |
| 164 | PREX1 | 10126 | 0.000784 | 0.446 | NO |
| 165 | GRK7 | 10543 | 0.00074 | 0.425 | NO |
| 166 | FOXO3 | 10779 | 0.000713 | 0.414 | NO |
| 167 | DOCK2 | 10896 | 7.00E-04 | 0.409 | NO |
| 168 | CCL3 | 11012 | 0.000689 | 0.404 | NO |
| 169 | CXCL16 | 11268 | 0.00066 | 0.392 | NO |
| 170 | CXCL14 | 11488 | 0.000639 | 0.381 | NO |
| 171 | ADRBK2 | 11740 | 0.000613 | 0.369 | NO |
| 172 | CCL22 | 11925 | 0.000593 | 0.36 | NO |
| 173 | CCL7 | 13034 | 0.000489 | 0.301 | NO |
| 174 | CCL13 | 14121 | 0.00039 | 0.243 | NO |
| 175 | CCL8 | 14912 | 0.000324 | 0.201 | NO |
| 176 | CCL4 | 15016 | 0.000316 | 0.196 | NO |
| 177 | CX3CR1 | 15063 | 0.000311 | 0.195 | NO |
| 178 | CCL17 | 15536 | 0.000274 | 0.17 | NO |
| 179 | CCL24 | 15537 | 0.000274 | 0.17 | NO |
| 180 | CCL3L1 | 15605 | 0.000269 | 0.167 | NO |
| 181 | CCL3L3 | 15711 | 0.000261 | 0.162 | NO |
| 182 | CCL14 | 16705 | 0.000176 | 0.109 | NO |
| 183 | CCL15 | 16988 | 0.000155 | 0.0938 | NO |
| 184 | CCL1 | 17112 | 0.000146 | 0.0874 | NO |
| 185 | CCL26 | 17364 | 0.000125 | 0.0742 | NO |
| 186 | CCL4L2 | 17605 | 0.000106 | 0.0614 | NO |

**Supplementary Table S5.** 11 glioma associated lncRNAs in the Lnc2Cancer database

| **Ensembl ID** | **LncRNA name** | **PubMed ID** | **Year** | **Title** |
| --- | --- | --- | --- | --- |
| ENSG00000130600 | H19 | 24466011 | 2014 | Long non-coding RNA H19 promotes glioma cell invasion by deriving miR-675. |
| ENSG00000177640 | CASC2 | 25446261 | 2014 | Long non-coding RNA CASC2 suppresses malignancy in human gliomas by miR-21. |
| ENSG00000214548 | MEG3 | 25645334 | 2015 | Altered expression of long non-coding RNAs during genotoxic stress-induced cell death in human glioma cells |
| ENSG00000228630 | HOTAIR | 24203894 | 2013 | HOTAIR, a cell cycle-associated long noncoding RNA and a strong predictor of survival, is preferentially expressed in classical and mesenchymal glioma. |
| ENSG00000234883 | MIR155HG | 25645334 | 2015 | Altered expression of long non-coding RNAs during genotoxic stress-induced cell death in human glioma cells |
| ENSG00000236824 | BC200 | 25645334 | 2015 | Altered expression of long non-coding RNAs during genotoxic stress-induced cell death in human glioma cells |
| ENSG00000241684 | ADAMTS9-AS2 | 24833086 | 2014 | A new tumor suppressor LncRNA ADAMTS9-AS2 is regulated by DNMT1 and inhibits migration of glioma cells. |
| ENSG00000245532 | NEAT1 | 26582084 | 2015 | Aberrant NEAT1 expression is associated with clinical outcome in high grade glioma patients. |
| ENSG00000245694 | CRNDE | 25813405 | 2015 | CRNDE, a long-noncoding RNA, promotes glioma cell growth and invasion through mTOR signaling. |
| ENSG00000251562 | MALAT1 | 26649728 | 2015 | Downregulation of lncRNA-MALAT1 Affects Proliferation and the Expression of Stemness Markers in Glioma Stem Cell Line SHG139S |
| ENSG00000253352 | TUG1 | 25645334 | 2015 | Altered expression of long non-coding RNAs during genotoxic stress-induced cell death in human glioma cells |

**Supplementary Table S6.** Nine pancreatic-cancer associated lncRNAs in the Lnc2Cancer database

| **Ensembl ID** | **LncRNA name** | **PubMed ID** | **Year** | **Title** |
| --- | --- | --- | --- | --- |
| ENSG00000222041 | LINC00152 | 25910082 | 2015 | Next-generation sequencing reveals novel differentially regulated mRNAs, lncRNAs, miRNAs, sdRNAs and a piRNA in pancreatic cancer. |
| ENSG00000228630 | HOTAIR | 22614017 | 2013 | HOTAIR is a negative prognostic factor and exhibits pro-oncogenic activity in pancreatic cancer. |
| ENSG00000234741 | GAS5 | 24026436 | 2013 | Downregulation of gas5 increases pancreatic cancer cell proliferation by regulating CDK6. |
| ENSG00000242767 | RP11-58D2.1 | 25755691 | 2015 | Genomic analysis of drug resistant pancreatic cancer cell line by combining long non-coding RNA and mRNA expression profling. |
| ENSG00000243766 | HOTTIP | 25912306 | 2015 | The long non-coding RNA HOTTIP enhances pancreatic cancer cell proliferation, survival and migration. |
| ENSG00000250222 | CTC-338M12.5 | 25755691 | 2015 | Genomic analysis of drug resistant pancreatic cancer cell line by combining long non-coding RNA and mRNA expression profling. |
| ENSG00000251164 | HULC | 25412939 | 2014 | Long noncoding RNA HULC is a novel biomarker of poor prognosis in patients with pancreatic cancer. |
| ENSG00000251562 | MALAT1 | 25481511 | 2014 | Overexpression of long non-coding RNA MALAT1 is correlated with clinical progression and unfavorable prognosis in pancreatic cancer. |
| ENSG00000259974 | LINC00261 | 25910082 | 2015 | Next-generation sequencing reveals novel differentially regulated mRNAs, lncRNAs, miRNAs, sdRNAs and a piRNA in pancreatic cancer. |

**Supplementary Table S7. Pathways identified by three methods (LncRNAs2Pathways,** **LncRNA2Function, Co-LncRNA) for the set of differentially expressed lncRNAs in prostate cancer.**

| **Pathways** | **LncRNAs2Pathways**  **(BH's FDR<0.01)** | **LncRNA2Function**  **(BH's FDR<0.01)** | **CO-LncRNA**  **Bonferroni adjusted p-value<0.01** |
| --- | --- | --- | --- |
| Oxidative phosphorylation | **√** |  |  |
| Ribosome | **√** |  |  |
| Proteasome | **√** |  |  |
| Chemokine signaling pathway | **√** | **√** |  |
| Cell cycle | **√** |  |  |
| Focal adhesion | **√** |  |  |
| Gap junction | **√** | **√** |  |
| Regulation of actin cytoskeleton | **√** |  |  |
| Melanogenesis | **√** |  | **√** |
| Alzheimer's disease | **√** |  |  |
| Parkinson's disease | **√** |  |  |
| Huntington's disease | **√** |  |  |
| Vibrio cholerae infection | **√** |  |  |
| Epithelial cell signaling in Helicobacter pylori infection | **√** |  |  |
| Pathogenic Escherichia coli infection | **√** |  |  |
| Pathways in cancer | **√** |  |  |
| Spliceosome | **√** |  |  |
| Cardiac muscle contraction | **√** |  |  |
| GnRH signaling pathway | **√** |  |  |
| Vasopressin-regulated water reabsorption | **√** |  |  |
| Amphetamine addiction |  | **√** |  |
| Calcium signaling pathway |  | **√** |  |
| Cocaine addiction |  | **√** |  |
| Dopaminergic synapse |  | **√** |  |
| GABAergic synapse |  | **√** |  |
| Gastric acid secretion |  | **√** |  |
| Glutamatergic synapse |  | **√** |  |
| Long-term potentiation |  | **√** |  |
| Morphine addiction |  | **√** |  |
| Natural killer cell mediated cytotoxicity |  | **√** |  |
| Neuroactive ligand-receptor interaction |  | **√** |  |
| Nicotine addiction |  | **√** |  |
| Osteoclast differentiation |  | **√** |  |
| Retrograde endocannabinoid signaling |  | **√** |  |
| Serotonergic synapse |  | **√** |  |
| Synaptic vesicle cycle |  | **√** |  |
| Fc Gamma R Mediated Phagocytosis |  |  | **√** |
| Oocyte Meiosis |  |  | **√** |

# Pathways which were exclusively identified by LncRNAs2Pathways were mark with red.

**Supplementary Table S8. Pathways identified by three methods (LncRNAs2Pathways, LncRNA2Function, Co-LncRNA) for the set of lncRNAs associated with glioma.**

| **Pathways** | **LncRNAs2Pathways**  **(BH's FDR<0.01)** | **LncRNA2Function**  **(BH's FDR<0.01)** | **CO-LncRNA**  **Bonferroni adjusted p-value<0.01** |
| --- | --- | --- | --- |
| Ribosome | √ |  | √ |
| MAPK signaling pathway | √ |  | √ |
| ErbB signaling pathway | √ |  |  |
| Cell cycle | √ |  |  |
| Oocyte meiosis | √ |  |  |
| Focal adhesion | √ |  |  |
| Long-term potentiation | √ |  |  |
| Neurotrophin signaling pathway | √ |  |  |
| Regulation of actin cytoskeleton | √ |  |  |
| Insulin signaling pathway | √ |  | √ |
| Huntington's disease | √ |  |  |
| Pathways in cancer | √ |  | √ |
| Glioma | √ |  |  |
| Prostate cancer | √ |  |  |
| Melanoma | √ |  |  |
| Chronic myeloid leukemia | √ |  |  |
| Chemokine signaling pathway | √ |  |  |
| Adherens junction | √ |  |  |
| T cell receptor signaling pathway | √ |  |  |
| B cell receptor signaling pathway | √ |  |  |
| Fc epsilon RI signaling pathway | √ |  |  |
| GnRH signaling pathway | √ |  |  |
| Alzheimer's disease | √ |  |  |
| Endometrial cancer | √ |  |  |
| Lysosome |  |  | √ |
| Rna Degradation |  |  | √ |
| Ubiquitin Mediated Proteolysis |  |  | √ |
| Aminoacyl Trna Biosynthesis |  |  | √ |
| Glycosylphosphatidylinositol Gpi Anchor Biosynthesis |  |  | √ |
| Basal Cell Carcinoma |  |  | √ |
| Other Glycan Degradation |  |  | √ |
| Mismatch Repair |  |  | √ |
| Homologous Recombination |  |  | √ |
| Colorectal Cancer |  |  | √ |
| Pyrimidine Metabolism |  |  | √ |
| Type Ii Diabetes Mellitus |  |  | √ |
| Peroxisome |  |  | √ |

# Pathways which were exclusively identified by LncRNAs2Pathways were mark with red.

**Supplementary Table S9. the network parameters of different coding-noncoding gene co-expression networks constructed by the different cutoffs of dataset number from two to nine.**

| **Cutoff** | **2** | **3** | **4** | **5** | **6** | **7** | **8** | **9** |
| --- | --- | --- | --- | --- | --- | --- | --- | --- |
| Number of nodes | 34424 | 27085 | 23820 | 6173 | 3943 | 1382 | 873 | 463 |
| Number of edges | 4903994 | 114476 | 69803 | 8002 | 4513 | 1578 | 1016 | 546 |
| Number of coding gene | 18779 | 16068 | 14761 | 5248 | 3330 | 1153 | 694 | 363 |
| Number of noncoding gene | 15645 | 11017 | 9059 | 925 | 613 | 229 | 179 | 100 |
| Number of coding-coding genes links | 2510918 | 85390 | 51419 | 6826 | 3781 | 1329 | 824 | 447 |
| Number of noncoding-coding genes links | 1457772 | 17794 | 11290 | 825 | 489 | 171 | 130 | 66 |
| Number of noncoding-noncoding genes links | 935304 | 11283 | 7094 | 351 | 243 | 78 | 62 | 33 |
| Network centralization | 0.09613 | 0.008365 | 0.008528 | 0.008653 | 0.01185 | 0.027311 | 0.037468 | 0.04035 |
| Clustering coefficient | 0.068198 | 0.085005 | 0.094655 | 0.308928 | 0.315261 | 0.331926 | 0.320654 | 0.309952 |
| Avg. number of neighbors | 284.9172 | 8.452428 | 5.860873 | 2.592581 | 2.28912 | 2.283647 | 2.327606 | 2.358531 |
| Parameter gamma in power-law | 1.2 | 2.261 | 2.363 | 2.195 | 2.052 | 1.874 | 1.763 | 1.697 |
| Fitted line R-squared in power law | 0.5568 | 0.9046 | 0.9242 | 0.9418 | 0.9414 | 0.9096 | 0.9168 | 0.9005 |

**REFERENCES**

**1 Kanehisa, M., Goto, S., Sato, Y., Furumichi, M. & Tanabe, M. KEGG for integration and interpretation of large-scale molecular data sets. *Nucleic Acids Res* 40, D109-114 (2012).**

**2 Kanehisa, M., Furumichi, M., Tanabe, M., Sato, Y. & Morishima, K. KEGG: new perspectives on genomes, pathways, diseases and drugs. *Nucleic Acids Res* 45, D353-D361 (2017).**
